# Supplementary material for: Boosting the efficiency of organic persistent room-temperature phosphorescence by intramolecular triplet-triplet energy transfer
Source: Nat Commun. 2019 Apr 8;10:1595. doi: 10.1038/s41467-019-09561-8 (PMC6453937; doi:10.1038/s41467-019-09561-8)
Supplement: Supplementary file 1 — Supplementary Information [file 41467_2019_9561_MOESM1_ESM.pdf]

Supplementary Information

Boosting the efficiency of organic persistent room temperature phosphorescence by intramolecular triplet-triplet energy transfer

Zhao et al.

Supplementary Methods

General Information

All the chemicals and reagents were purchased from Aldrich. All the molecules synthesized were purified by column chromatography and recrystallization from dichloromethane and hexane for three times, and fully characterized by ^{1}H NMR, ^{13}C NMR and high resolution mass spectroscopies and elementary analysis.

^{1}H and ^{13}C NMR spectra were recorded on a Bruker AV 400 Spectrometer at 400 and 100 MHz in $CDCl_3$, respectively. Tetramethylsilane was used as the internal standard. High-resolution mass spectra (HRMS) were recorded on a GCT premier CAB048 mass spectrometer operating in MALDI-TOF mode. Elementary analysis was performed on a Thermo Finnigan Flash EA1112. Gel filtration chromatography was performed using a ZORBAX SB-C18 column (Agilent) conjugated to an Agilent 1260 Infinite HPLC system. Before running, each sample was purified via 0.22 μm filter to remove any aggregates. The flow rate was fixed at 1.0 mL/min, the injection volume was 20 μL and each sample was run for 6 min. The absorption wavelength used was set at 330 nm. 100 % percent of acetonitrile was used as the running buffer. The photoluminescence spectra, lifetime, time-resolved excitation spectra, steady state and time-resolved emission spectra, temperature dependent photoluminescence spectra and absolute luminescence quantum yield were measured on a Edinburgh FLSP 980 fluorescence spectrophotometer equipped with a xenon arc lamp (Xe900), a microsecond flash-lamp (uF900), a picosecond pulsed diode laser (EPL-375), a closed cycle cryostate (CS202*I-DMX-1SS, Advanced Research Systems) and an integrating sphere (0.1 nm step size, 0.3 second integration time, 5 repeats), respectively. Mean decay times (τ_P) were obtained from individual lifetimes τ_i and amplitudes a_i of multi-exponential evaluation. Powder X-Ray diffraction patterns were performed on an X'Pert PRO MPD diffractometer with Cu Kα radiation (λ = 1.5418 Å) at 25 °C (scan range: 4.5−50°). Single crystal data was collected on a Bruker Smart APEXII CCD diffractometer using graphite monochromated Cu Kα radiation (λ = 1.54178 Å). The photos were recorded by a Cannon EOS 60D with the same parameters.

Recrystallization

All the crystalline samples were obtained from slowly evaporative crystallization using hexane/dichloromethane mixture (2:1, v/v). To further check the purity of the solid samples, all the solid samples were dissolved in 100 % percent of acetonitrile and got sample solutions (50 μM), then run the HPLC.

Preparation of blended powders

For the optical measurements of blended powders, we placed a mixture of CZ (1 mmol) and each other fragment (1 mmol) in a glass tube and heated it up to 150 °C for 10 s. After melting, the substrate was cooled rapidly to room temperature.

Synthesis

Molecules used in this study:

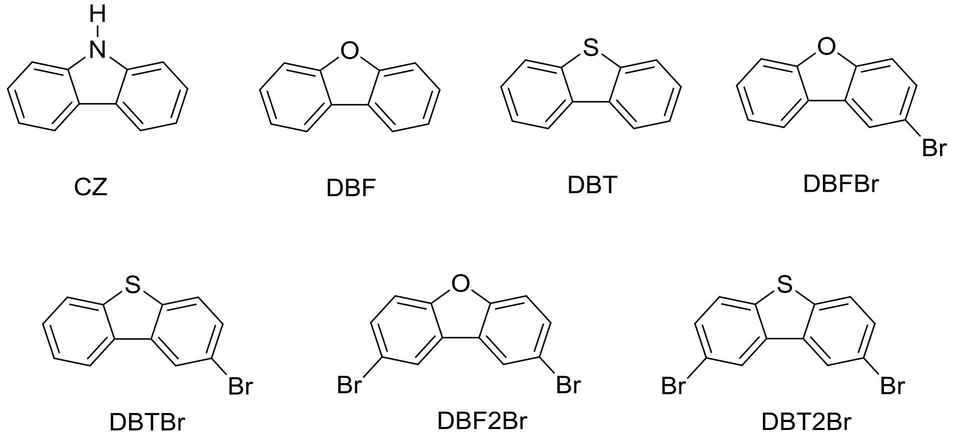

9-H-carbazole (CZ), dibenzofuran (DBF) and dibenzothiophen (DBT) were purchased from Sigma-Aldrich and further purified by column chromatography and recrystallization from dichloromethane and hexane for three times. 2-bromodibenzofuran (DBFBr), 2,8-dibromodibenzofuran (DBF2Br), 2-bromodibenzothiophene (DBTBr) and 2,8-dibromodibenzofuran (DBT2Br) were synthesized according to the reported synthetic route, and then recrystallized from dichloromethane and hexane for five times. All the above compounds were fully characterized by ^{1}H NMR and their purity were further checked by HPLC.

9-(dibenzo[b,d]furan-2-yl)-9H-carbazole (CZ-DBF)

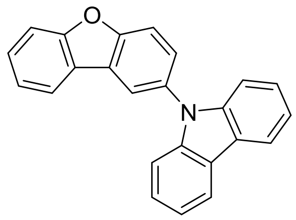

2-bromodibenzofuran (1.5 g, 6.07 mmol), 9-H-carbazole (1.12 g, 6.68 mmol), CuI (1.7 g, 9.11 mmol) and K_2CO_3 (2.5 g, 18.2 mmol) were added into a 100 mL flask. DMAc (50 mL) was then added into the flask. The solution was kept at 180° C and stirred under nitrogen for 24 h. After cooling, the solution was poured into water (200 mL), then extracted with excess CH_2Cl_2, dried by MgSO4, and purified using a silica gel column by using pure hexane as eluent. The obtained product was a white solid (0.81 g, 40% yield). ^{1}H NMR (400 MHz, $CDCl_3$): δ 8.20 (s, 1H), 8.178 (s, 1H), 8.12, 8.12(d, J = 2.0 Hz, 1H), 7.96-7.94 (m, 1H), 7.80, 7.78 (d, J = 8.4 Hz, 1H), 7.67, 7.65 (d, J = 8.4 Hz, 1H), 7.64-7.61 (m, 1H), 7.56-7.52 (m, 1H), 7.45-7.34 (m, 5H) and 7.31 (m, 2H). ^{13}C NMR (100 MHz, $CDCl_3$): δ 157.2, 155.3, 141.8, 132.8, 128.1, 126.8, 126.2, 125.9, 124.0, 123.4, 123.3, 121.2, 120.6, 120.1, 120.0, 113.1, 112.2, 109.9 . HRMS (MALDI-TOF, *m/z*): calcd. for $C_{24}H_{15}NO$, 333.1154. Found, 333.1174. Elemental analysis (calcd., found for $C_{24}H_{15}NO$): C (86.46, 86.52), H (4.54, 4.59).

9-(dibenzo[b,d]thiophen-2-yl)-9H-carbazole (CZ-DBT)

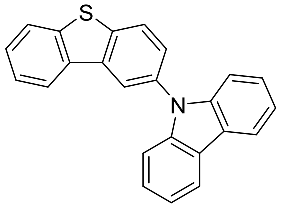

Following the same synthetic procedure for DBF-CZ, the reaction of 2-bromodibenzo[b,d]thiophene (1.5 g, 5.7 mmol), 9-H-carbazole (1.05 g, 6.27 mmol) , CuI (1.6 g, 8.55 mmol) and K_2CO_3 (2.4 g, 17.1 mmol) at 180° C and stirred under nitrogen for 24 h afforded the product as white solid. Yield: 46%. ^{1}H NMR (400 MHz, $CDCl_3$): δ: 8.34, 8.33 (d, J = 4.0 Hz, 1H), 8.20, 8.18 (d, J = 8 Hz, 2H), 8.13, 8.11 (d, J =8.0 Hz, 1H), 8.08, 8.06 (d, J = 8.0 Hz, 1H), 7.93, 7.91 (d, J = 8.0 Hz, 1H), 7.65-7.63 (m, 1H), 7.54-7.47 (m, 2H), 7.43, 7.42 (d, J = 4.0 Hz, 4H) and 7.33-7.29 (m, 2H). ^{13}C NMR (100 MHz, $CDCl_3$): δ: 140.7, 139.6, 137.9, 136.5, 134.4, 133.9, 126.8, 125.4, 125.3, 124.1, 123.5, 122.8, 122.4, 121.3, 120.0, 119.4 and 109.1. HRMS (MALDI-TOF, *m/z*): calcd for $C_{24}H_{15}NS$, 349.0925. Found, 349.0918 Elemental analysis (calcd., found for $C_{24}H_{15}NS$): C (82.49, 82.45), H (4.33, 4.29).

9-(8-bromodibenzo[b,d]furan-2-yl)-9H-carbazole (CZ-DBFBr)

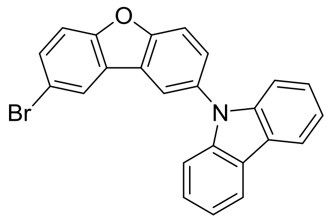

Following the same synthetic procedure for DBF-CZ, the reaction of 2,8-dibromodibenzo[b,d]furan (5.24 g, 16.1 mmol), 9-H-carbazole (3.23 g, 19.3 mmol) , CuI (1.53 g, 8.05 mmol) and K_2CO_3 (6.6 g, 48.3 mmol) at 180°C and stirred under nitrogen for 24 h afforded the product as white solid. Yield: 36%. ^{1}H NMR (400 MHz, $CDCl_3$): δ: 8.19 (s, 1H), 8.17 (s, 1H), 8.09 – 8.07 (m, 2H), 7.80, 7.78 (d, J = 8.0 Hz, 1H), 7.67 – 7.62 (m, 2H), 7.55, 7.53 (d, J = 8.0 Hz, 1H), 7.45 – 7.30 (m, 6H). ^{13}C NMR (100 MHz, $CDCl_3$): δ: 155.9, 155.7, 141.6, 133.2, 130.9, 127.6, 126.3, 126.0, 124.9, 124.0, 123.5, 120.6, 120.2, 120.1, 116.2, 113.7, 113.4 and 109.8. HRMS (MALDI-TOF, *m/z*): calcd for $BrC_{24}H_{14}NO$, 411.0259. Found, 411.0240. Elemental analysis (calcd., found for $C_{24}H_{15}NS$): C (69.92, 69.87), H (3.42, 3.46).

9-(8-bromodibenzo[b,d]thiophen-2-yl)-9H-carbazole (CZ-DBTBr)

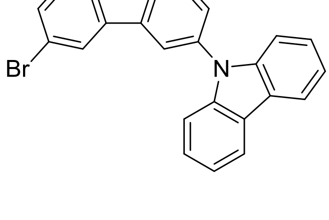

Following the same synthetic procedure for DBF-CZ, the reaction of 2,8-dibromodibenzo[b,d]furan (5.24 g, 15.3 mmol), 9-H-carbazole (3.07 g, 18.4 mmol) , CuI (1.50 g, 7.65 mmol) and K_2CO_3 (6.34 g, 45.9 mmol) at 180°C and stirred under nitrogen for 24 h afforded the product as white solid. Yield: 42%. ^1H NMR (400 MHz, $CDCl_3$): δ: 8.3 (d, J = 1.6 Hz, 1H), 8.26, 8.25 (d, J = 1.6 Hz, 1H), 8.20 (s, 1H), 8.18 (s, 1H), 8.08 – 8.06 (d, J = 8.8 Hz, 1H), 7.80, 7.78 (d, J = 8.4 Hz, 1H), 7.70 – 7.67 (m, 1H), 7.63 – 7.60 (m, 1H), 7.44 – 7.42 (m, 4H) and 7.34 – 7.31 (m, 2H) . ^{13}C NMR (100 MHz, $CDCl_3$): δ: 140.6, 138.3, 138.2, 136.2, 135.3, 134.3, 129.6, 126.0, 125.5, 124.2, 123.7, 123.6, 122.8, 119.9, 119.8, 119.5, 118.0). HRMS (MALDI-TOF, *m*/*z*): calcd for $BrC_{24}H_{14}NS$, 427.0030. Found, 427.0039. Elemental analysis (calcd., found for $C_{24}H_{15}NS$): C (67.30, 67.38), H (3.29, 3.23).

Supplementary Figures

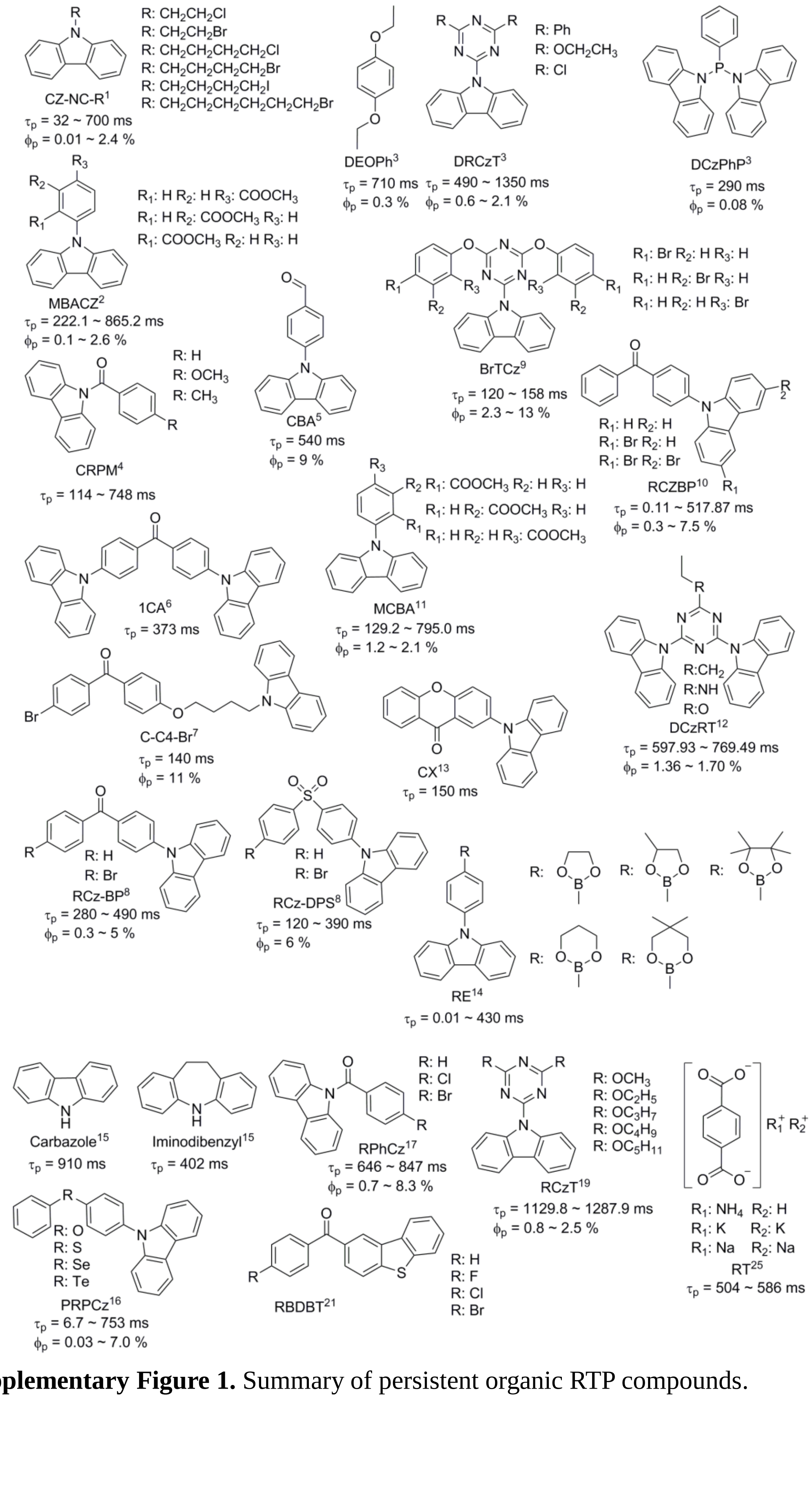

Supplementary Figure 1. Summary of persistent organic RTP compounds.

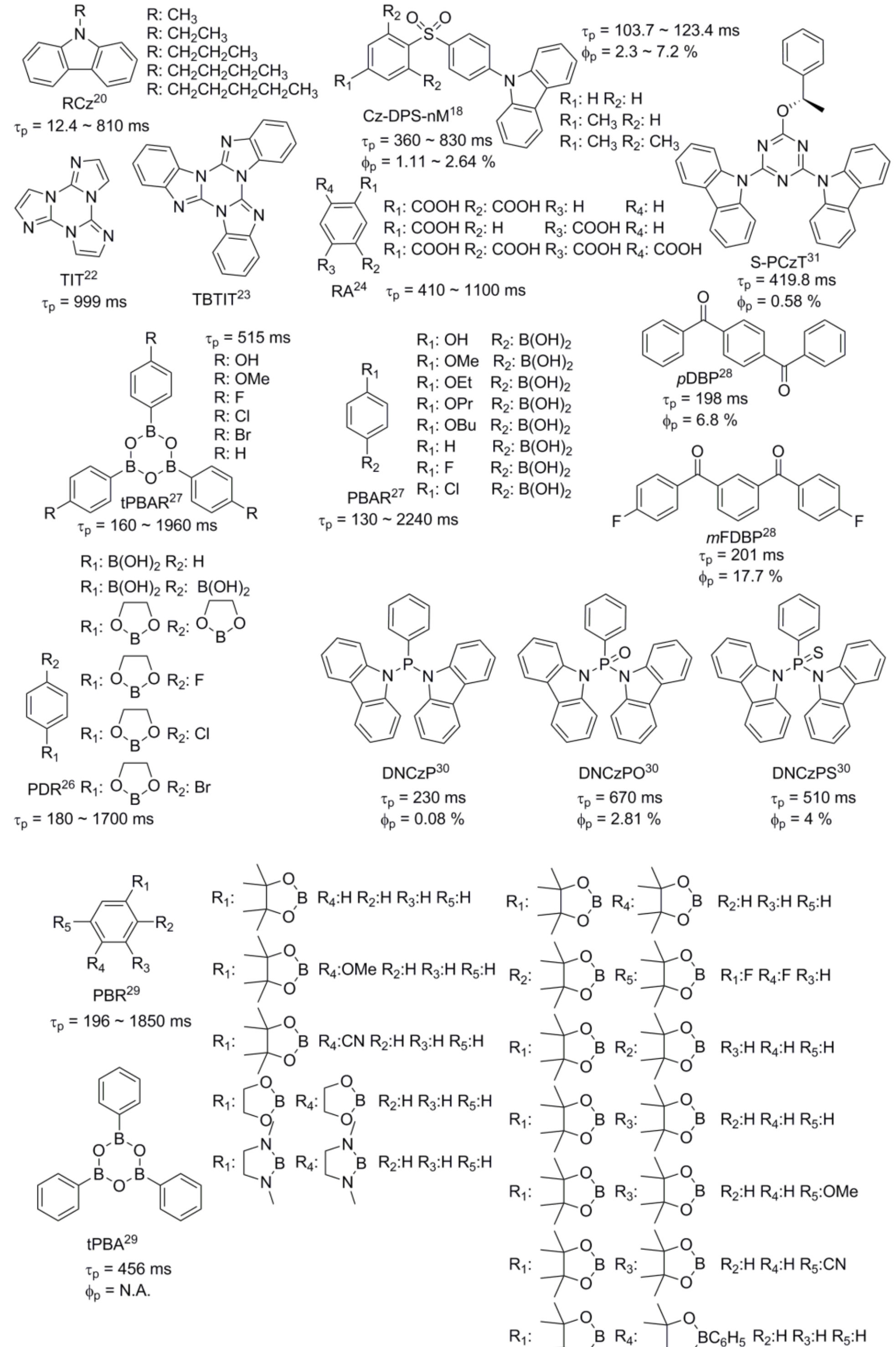

Supplementary Figure 2. Summary of persistent organic RTP compounds.

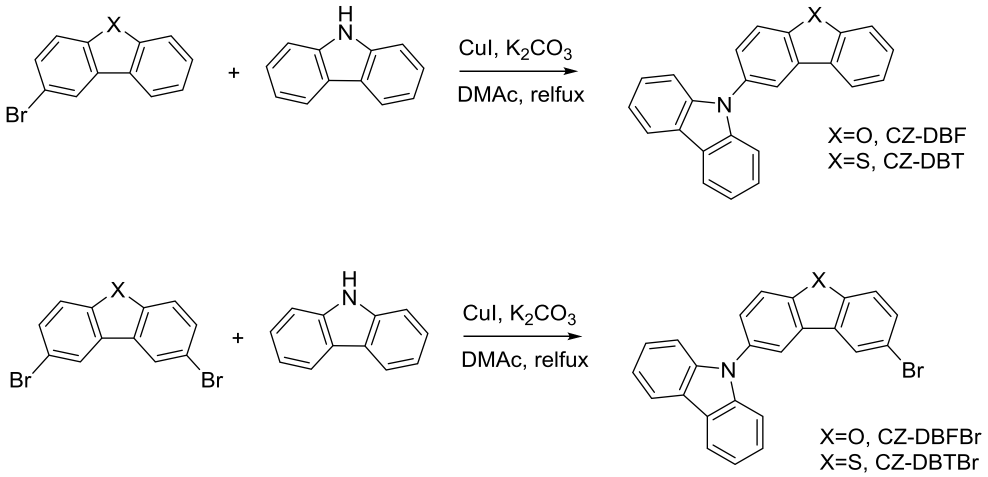

Supplementary Figure 3. Synthetic routes for OPRTP compounds.

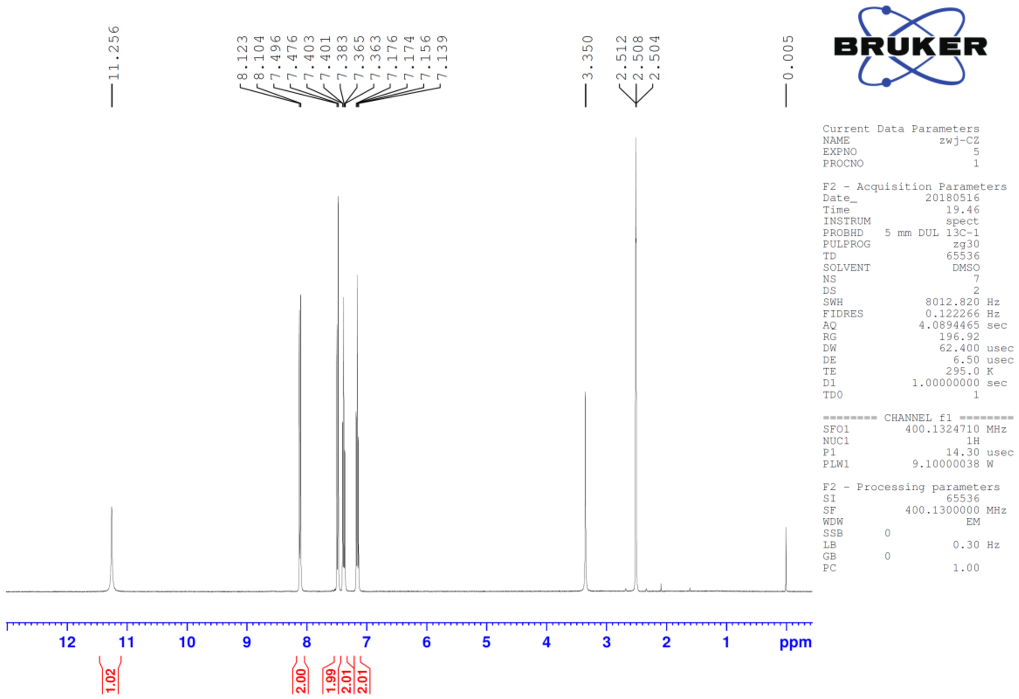

Supplementary Figure 4. ^{1}H NMR spectrum of CZ.

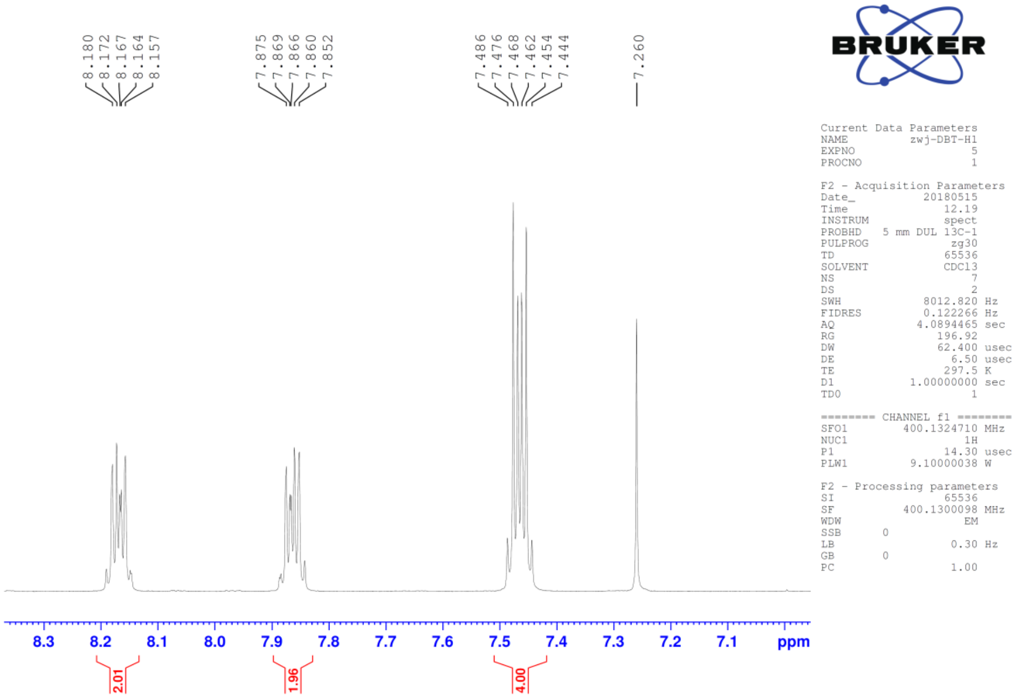

Supplementary Figure 5. ^{1}H NMR spectrum of DBT.

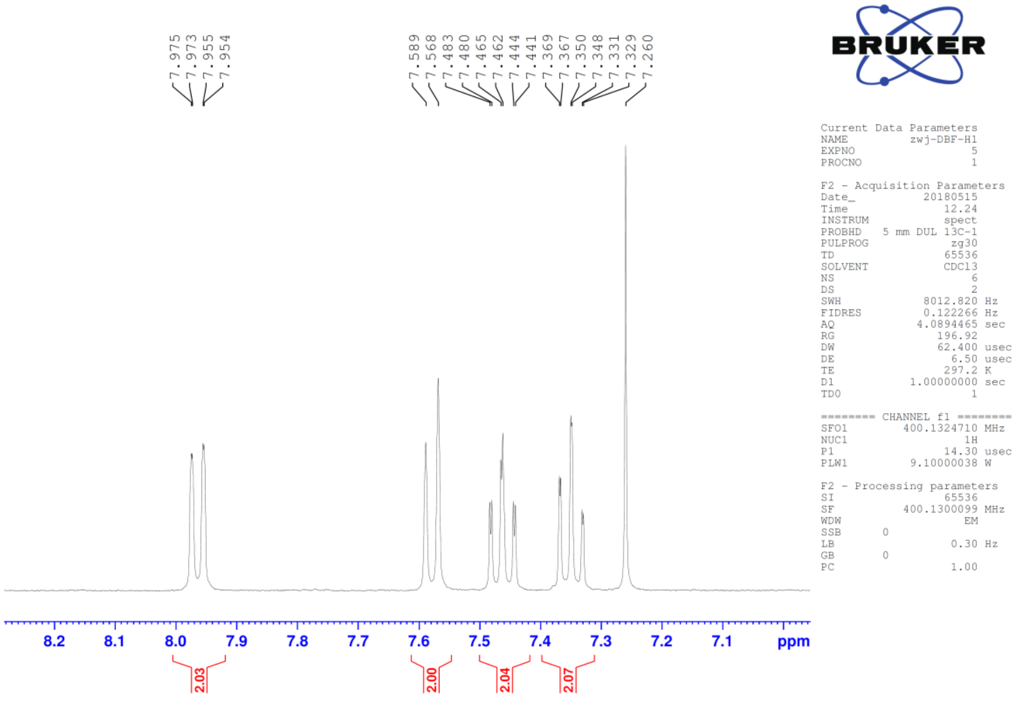

Supplementary Figure 6. ^{1}H NMR spectrum of BDF.

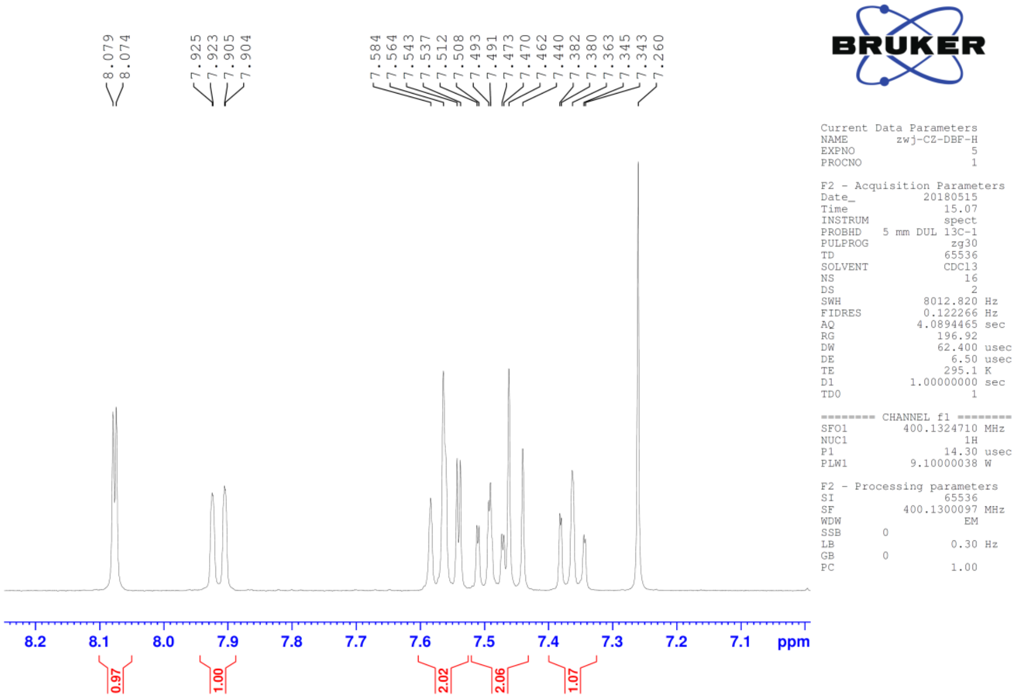

Supplementary Figure 7. ^{1}H NMR spectrum of BDFBr.

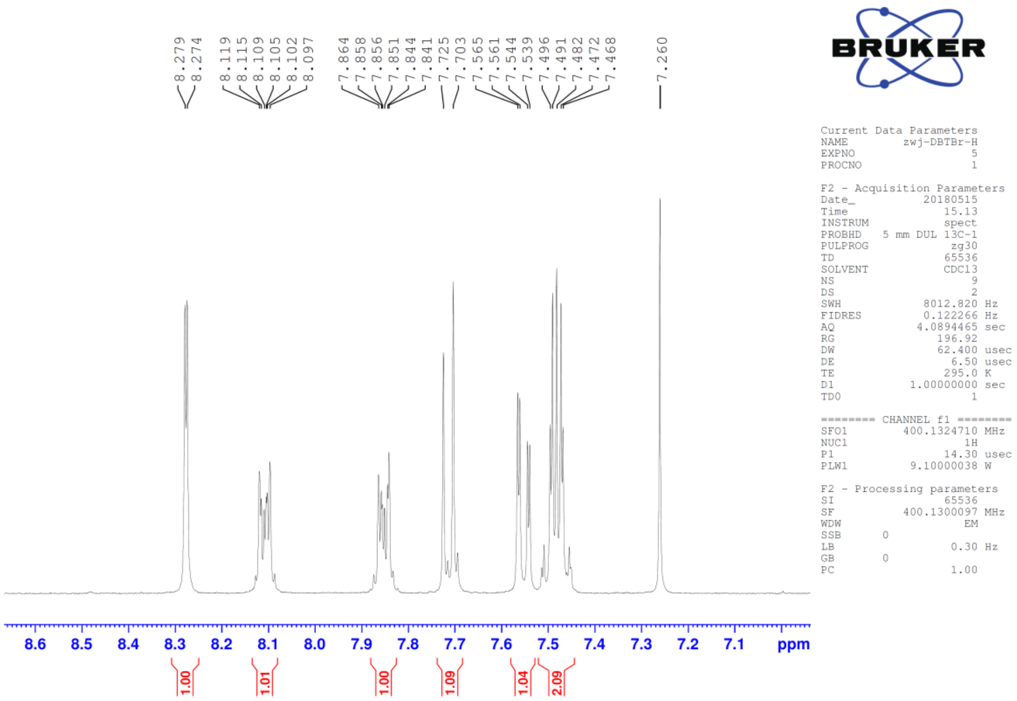

Supplementary Figure 8. ^{1}H NMR spectrum of BDTBr.

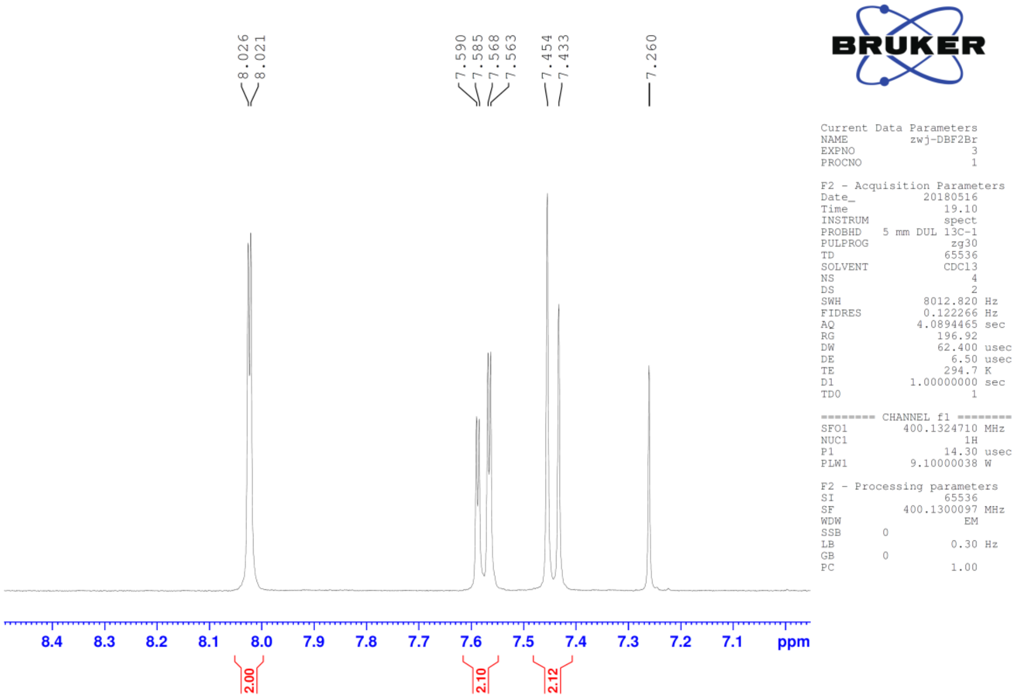

Supplementary Figure 9. ^{1}H NMR spectrum of BDF2Br.

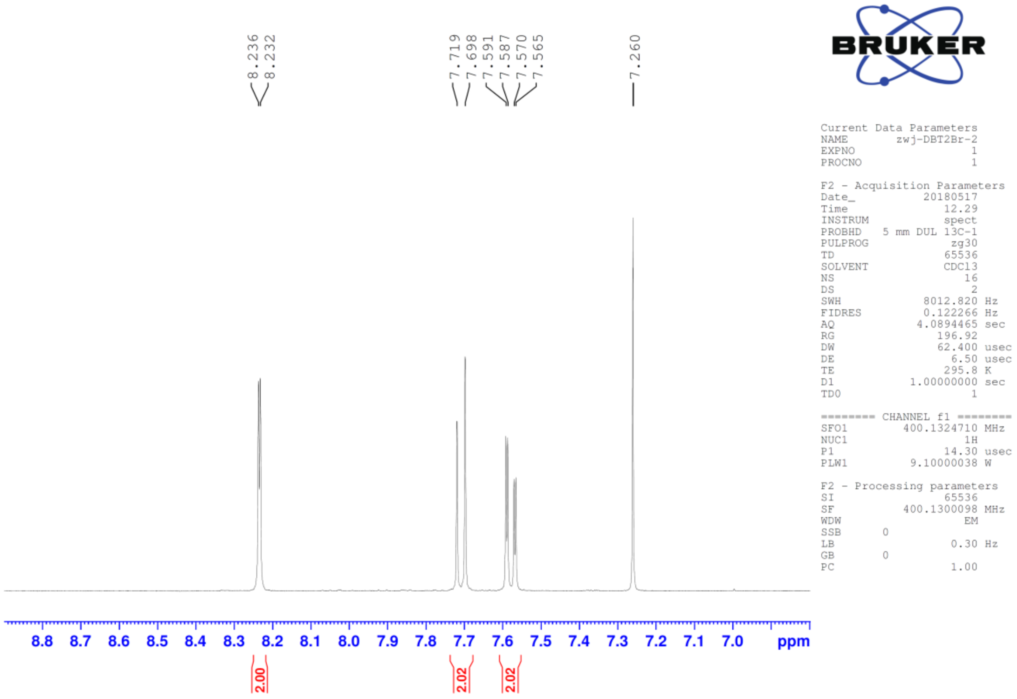

Supplementary Figure 10. ^{1}H NMR spectrum of BDT2Br.

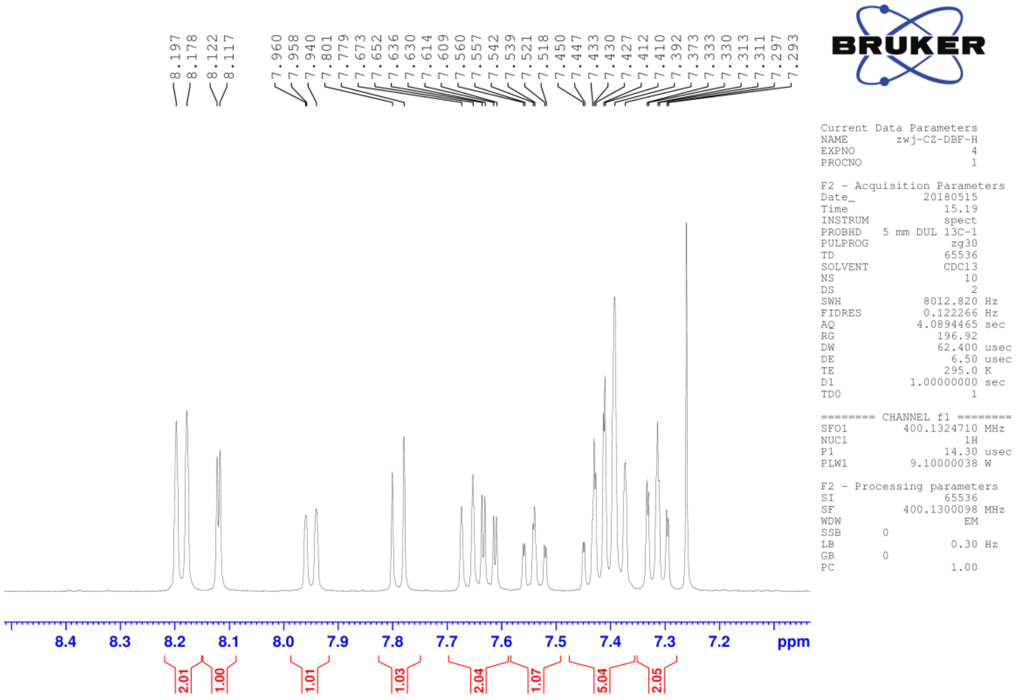

Supplementary Figure 11. ^{1}H NMR spectrum of CZ-DBF.

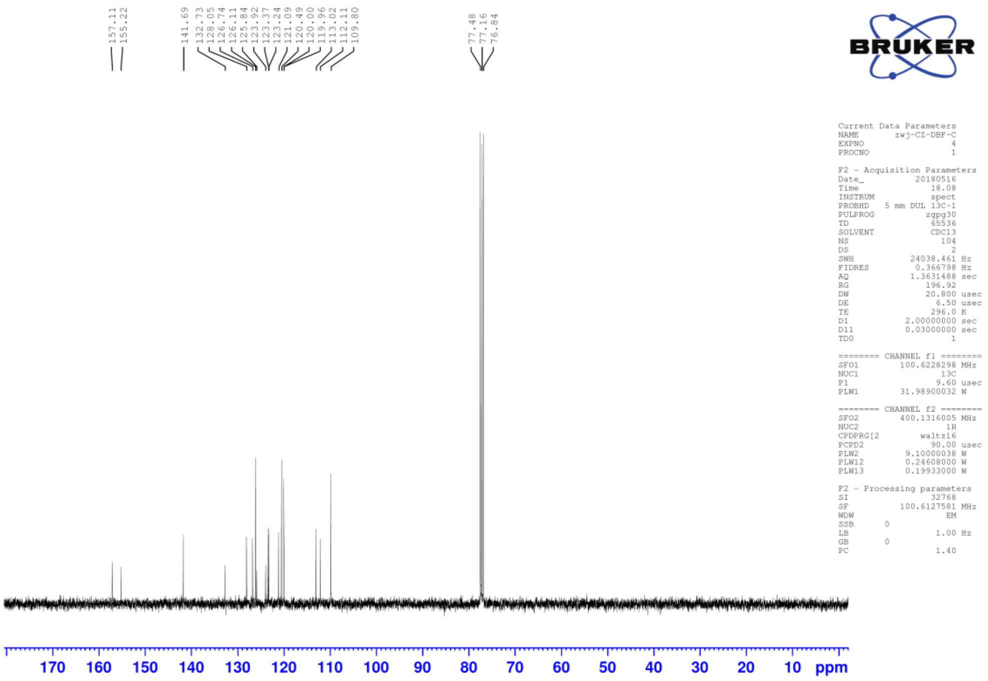

Supplementary Figure 12. ^{13}C NMR spectrum of CZ-DBF.

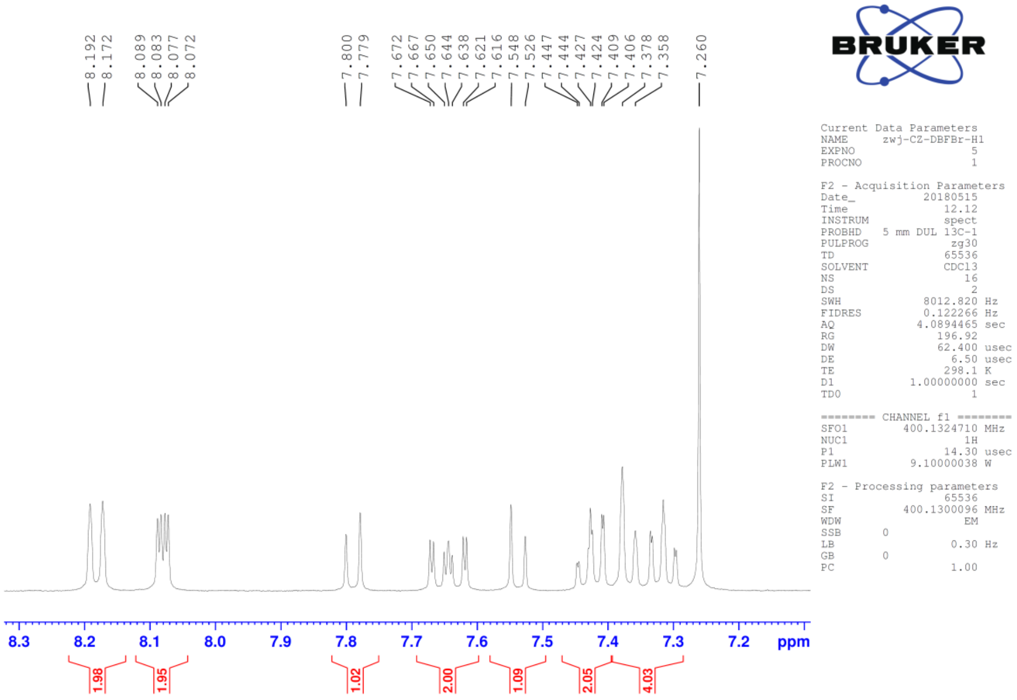

Supplementary Figure 13. ^{1}H NMR spectrum of CZ-DBFBr.

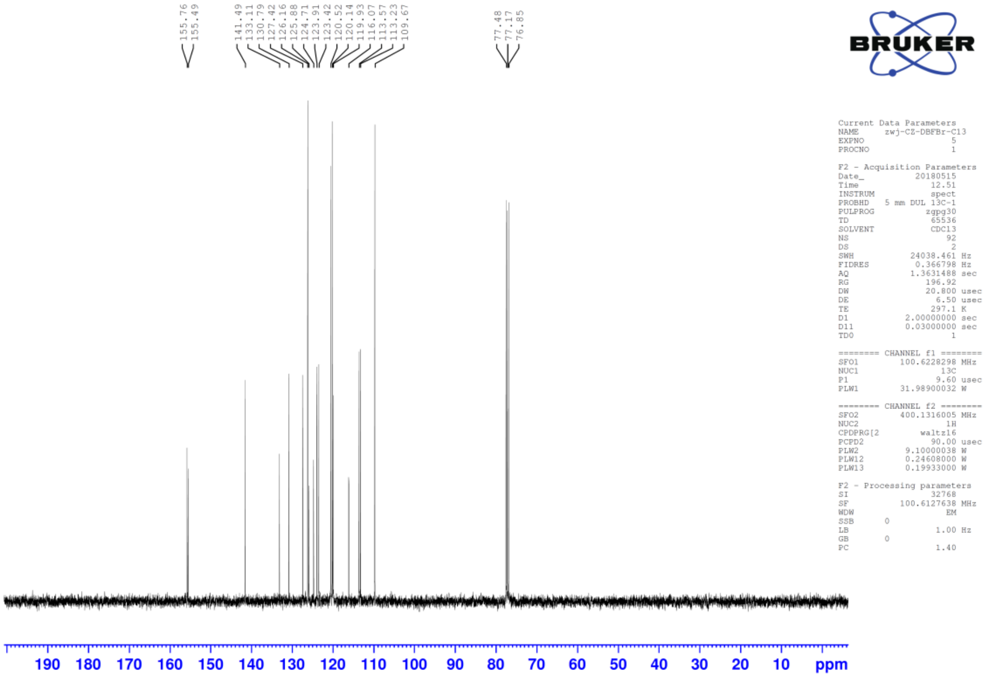

Supplementary Figure 14. ^{13}C NMR spectrum of CZ-DBFBr.

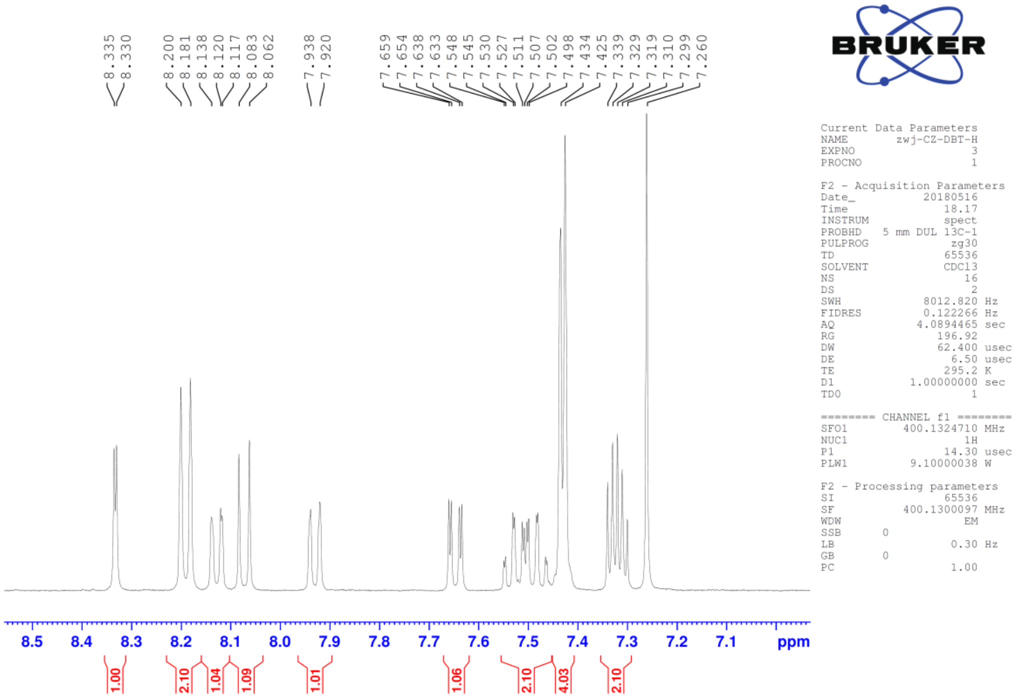

Supplementary Figure 15. ^{1}H NMR spectrum of CZ-DBT.

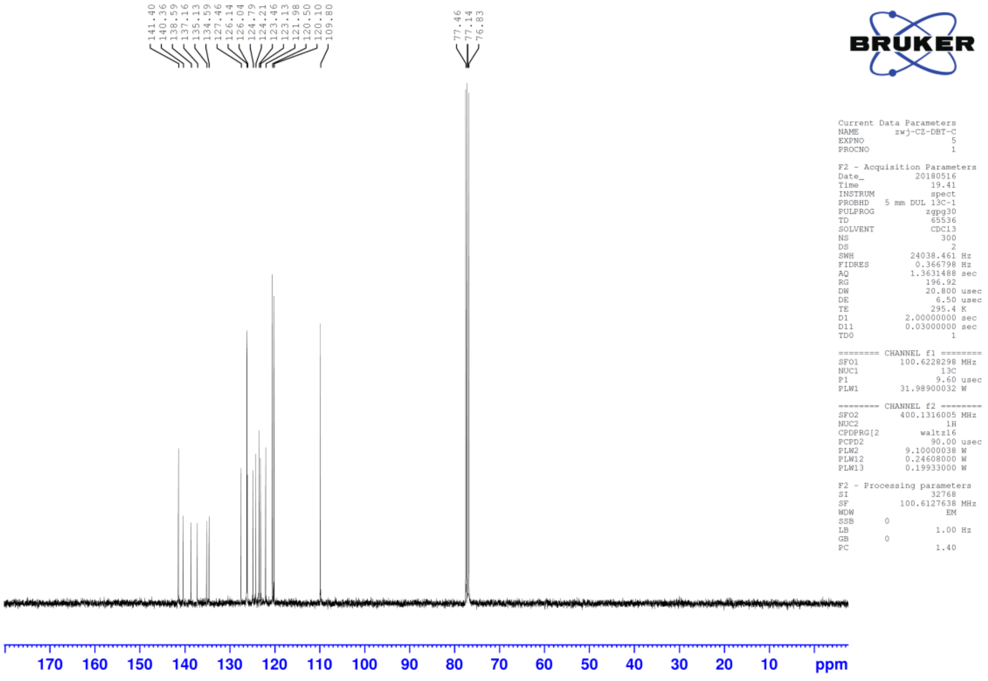

Supplementary Figure 16. ^{13}C NMR spectrum of CZ-DBT.

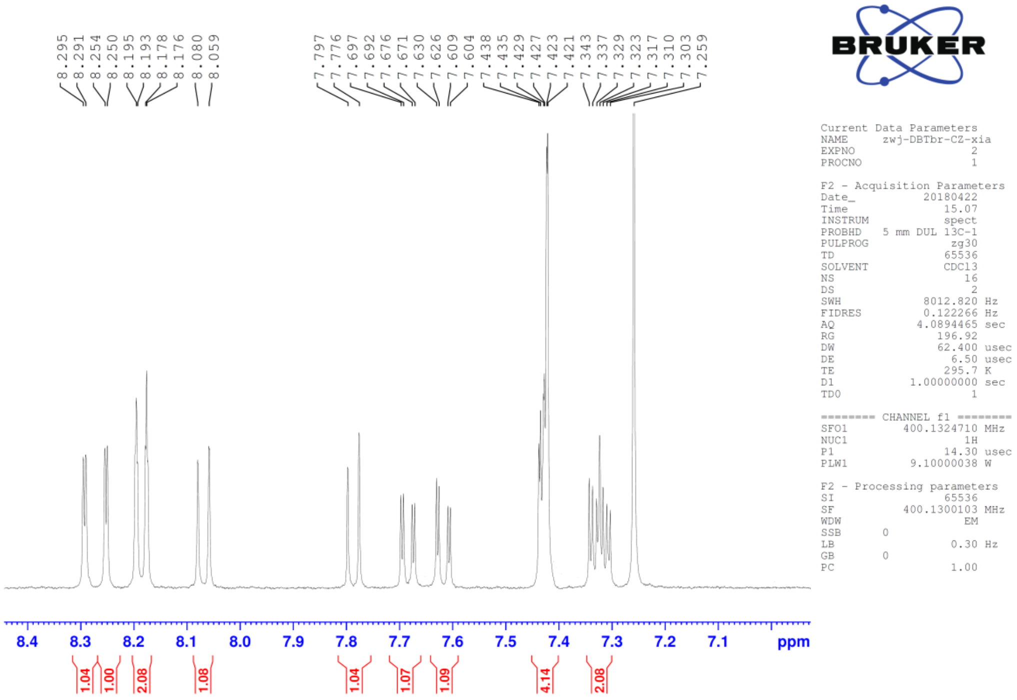

Supplementary Figure 17. ^{1}H NMR spectrum of CZ-DBTBr.

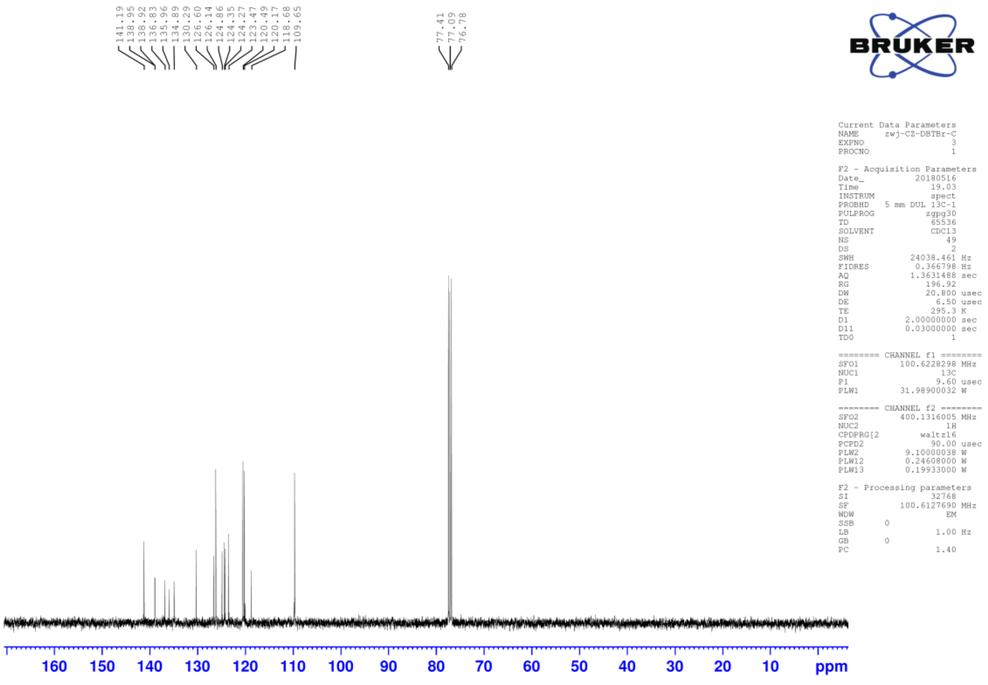

Supplementary Figure 18. ^{13}C NMR spectrum of CZ-DBTBr.

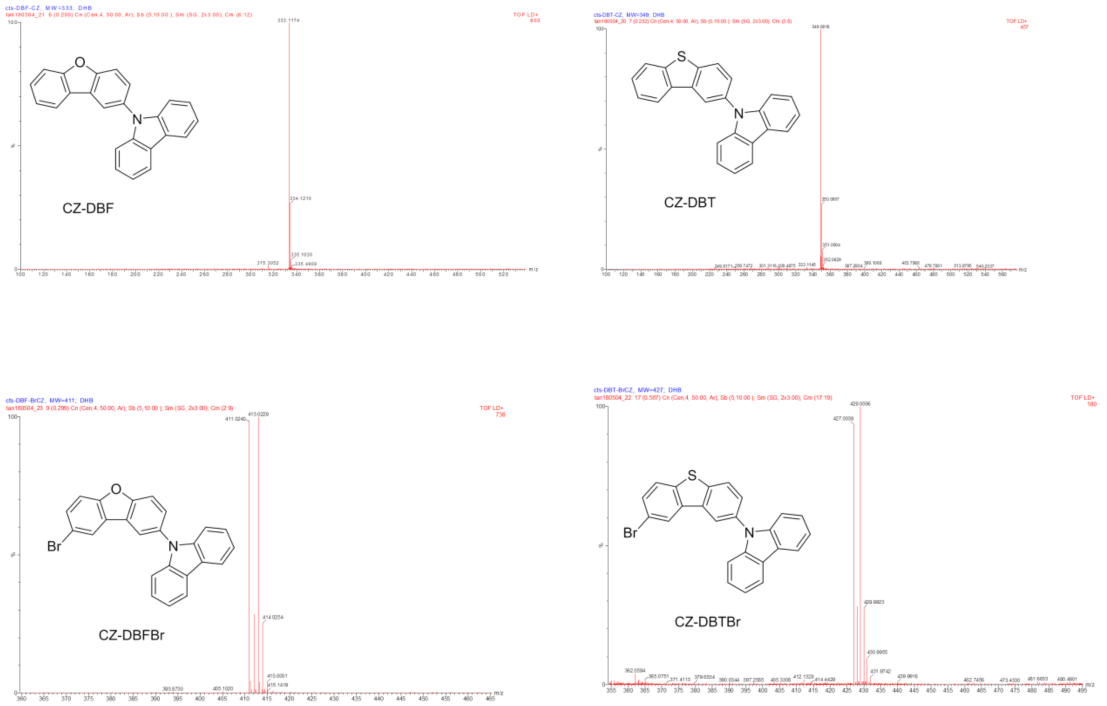

Supplementary Figure 19. HRMS spectra of OPRTP compounds.

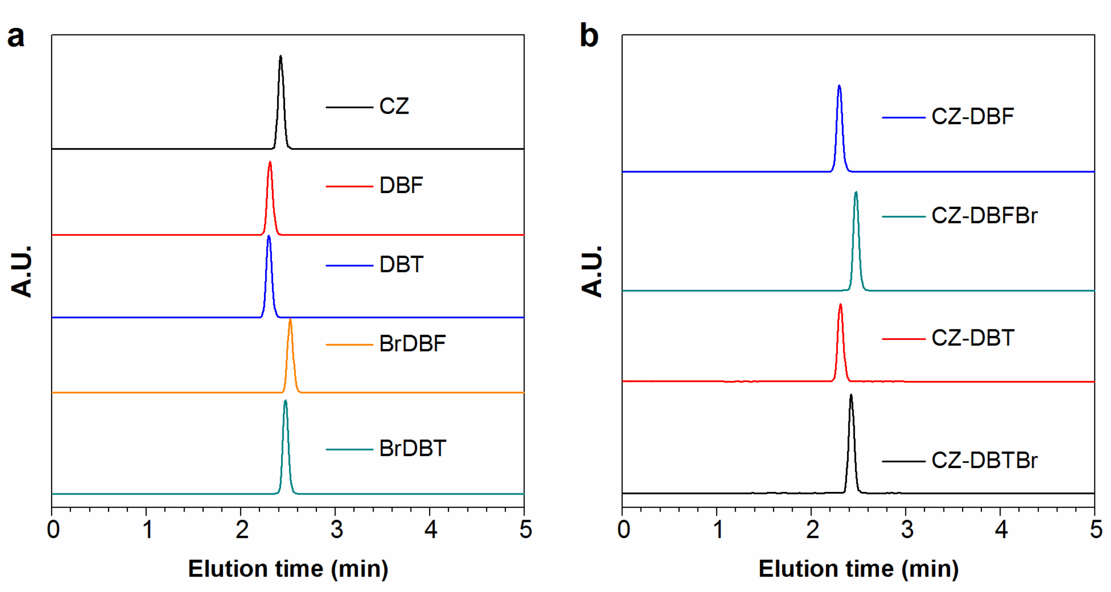

Supplementary Figure 20. High-performance liquid chromatogram spectra. HPLC of (**a**) CZ, DBF, DBT, DBFBr and DBTBr; (**b**) CZ-DBF, CZ-DBFBr, CZ-DBT and CZ-DBTBr in acetonitrile solution (50 μM).

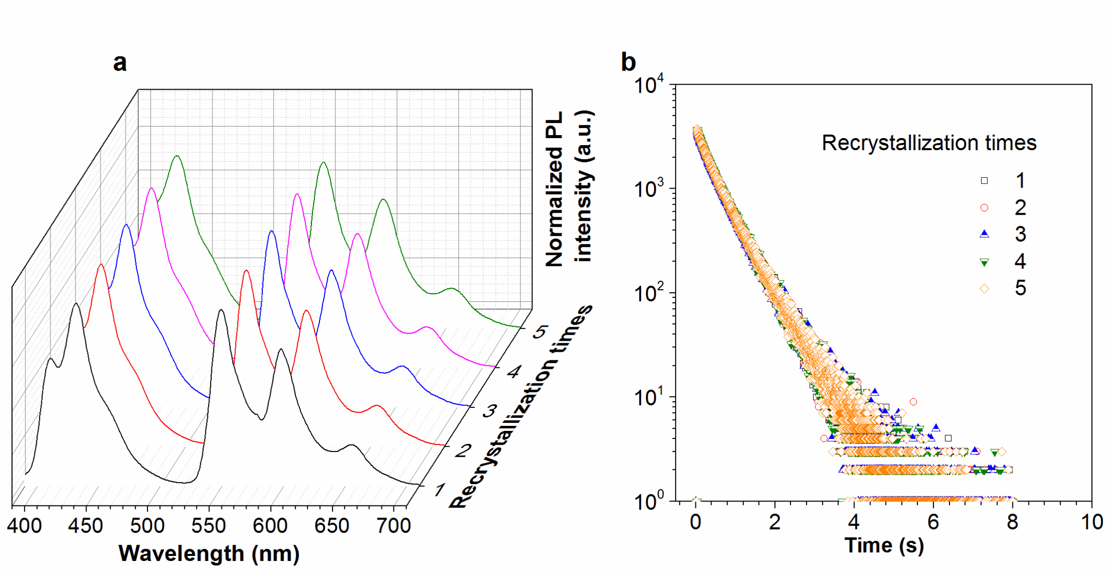

Supplementary Figure 21. Photophysical properties during recrystallization. (**a**) PL spectra and (**b**) time resolved PL decay curves of crystalline CZ-DBFBr after each time of recrystallization (5 times). Excited wavelength: 365 nm.

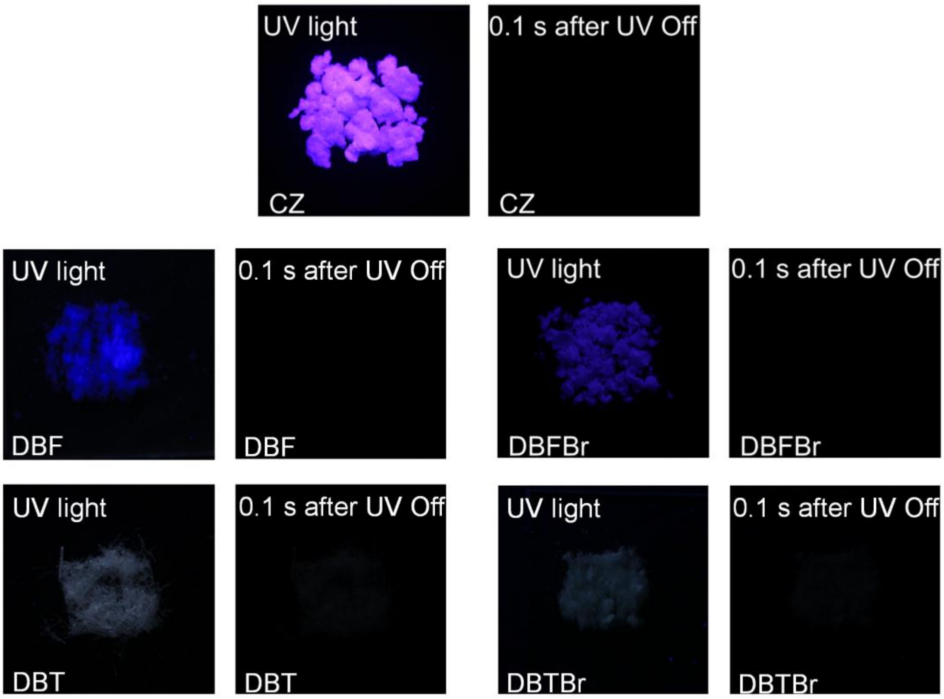

Supplementary Figure 22. Photographs of crystalline model fragments. Photographs of crystalline CZ, DBF, DBFBr, DBT and DBTBr taken before and after the removal of excitation source of UV light at ambient conditions. Excitation wavelength: 365 nm.

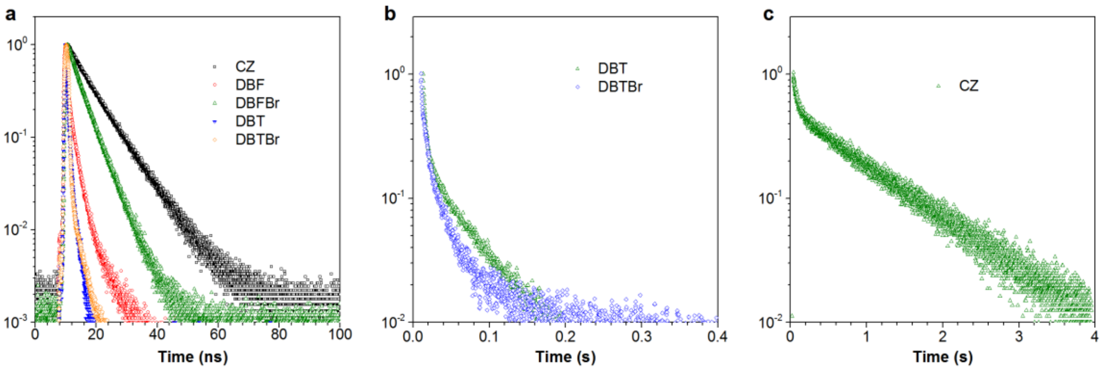

Supplementary Figure 23. PL decay curves of crystalline model fragments. (**a**) Nanosecond-scale PL decay curves of CZ, DBF, DBFBr, DBT and DBTBr. (**b**, **c**) Second-scale PL decay curves of DBT, DBTBr (**b**) and CZ (**c**) at 300 K.

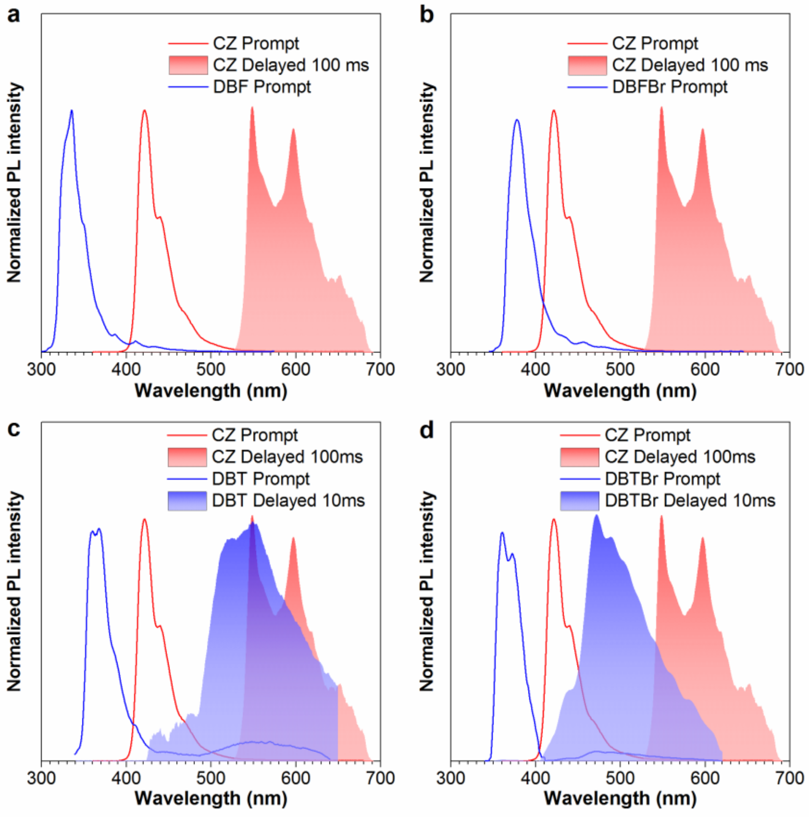

Supplementary Figure 24. PL spectra of crystalline model fragments at 300 K. The prompt (solid line) and delayed (red zone) PL spectra of crystalline CZ and DBF (**a**), DBFBr (**b**), DBT (**c**) and DBTBr (**d**) at 300 K. The excitation wavelength: CZ, 365 nm; DBF, 295 nm; DBFBr, DBT and DBTBr, 330 nm.

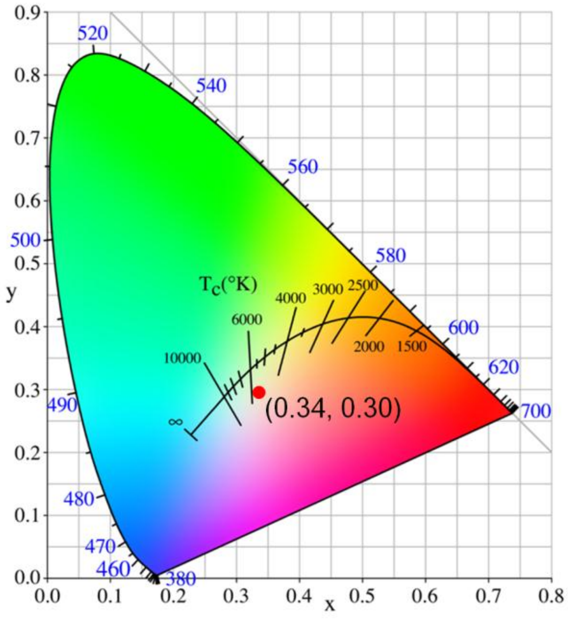

Supplementary Figure 25. CIE 1931 coordinates of CZ-DBFBr at 300 K.

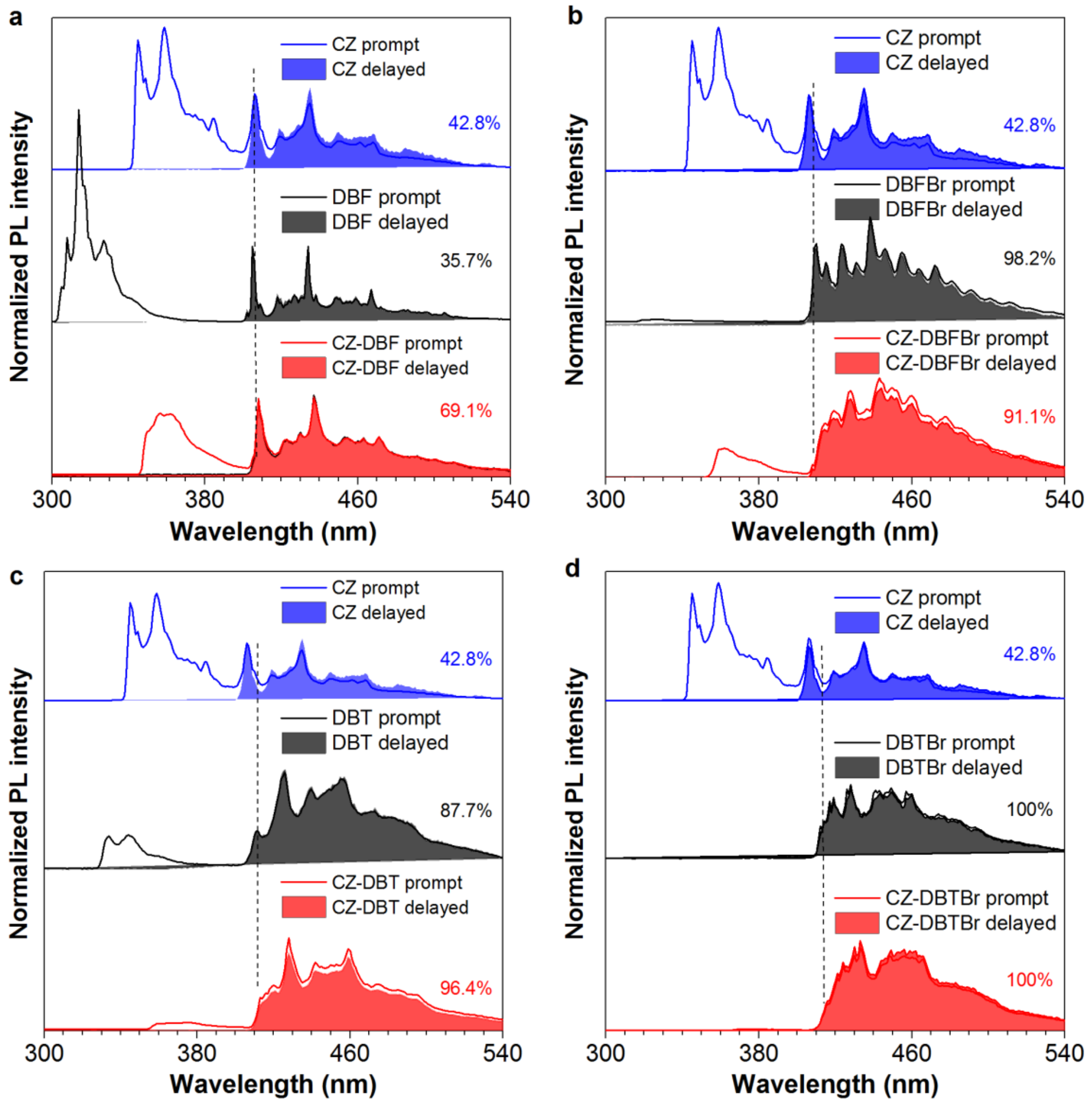

Supplementary Figure 26. PL spectra of RTP compounds in solutions at 77 K. The prompt (line) and delayed (color zone) PL spectra of the 2-methyl-tetrahydrofuran solutions of CZ, DBF and CZ-DBF (**a**), DBFBr and CZ-DBFBr (**b**), DBT and CZ-DBT (**c**), DBTBr and CZ-DBTBr (**d**) at 77 K with the proportion of phosphorescence inset. Excitation wavelength: 290 nm.

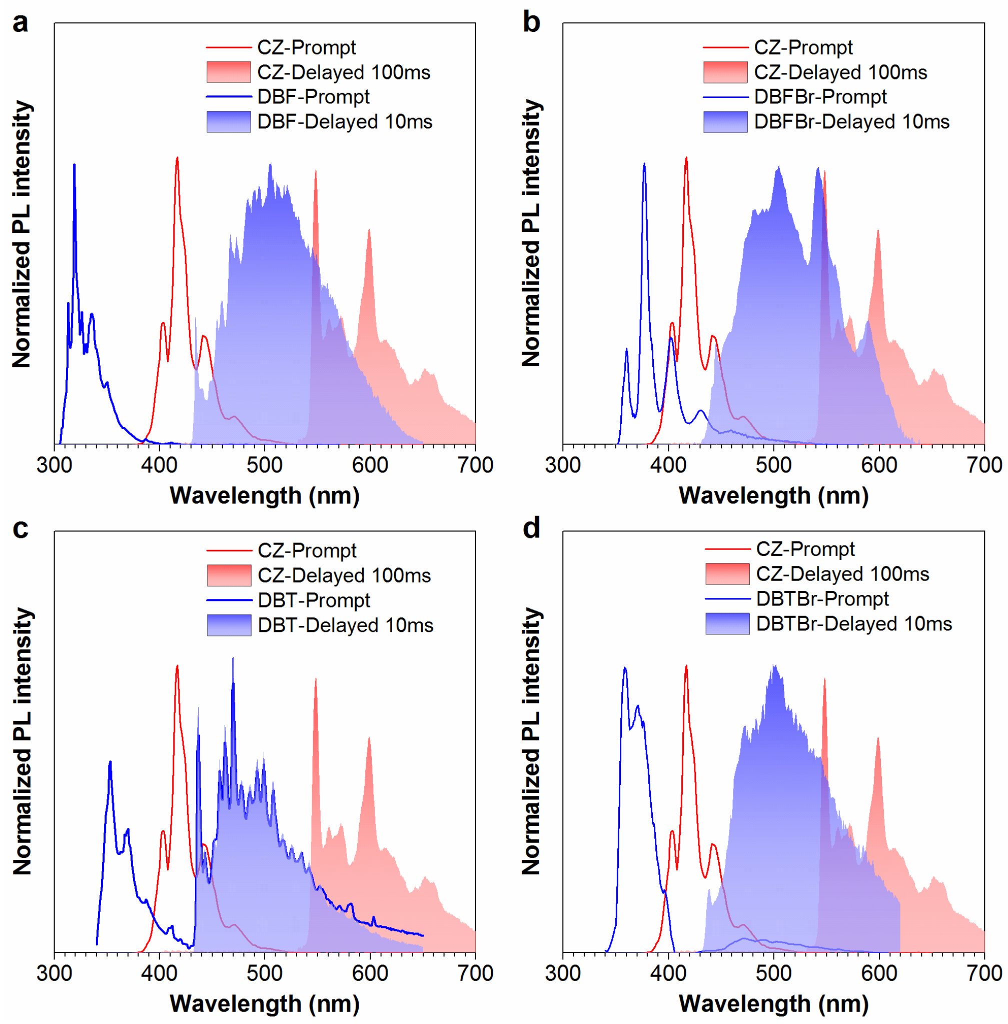

Supplementary Figure 27. PL spectra of RTP compounds in solid states at 4 K. The prompt (solid line) and delayed (color zone) PL spectra of the crystalline powders of CZ and DBF (**a**), DBFBr (**b**), DBT (**c**) and DBTBr (**d**) at 4 K. The excitation wavelength: CZ, 365 nm; DBF, 295 nm; DBFBr, DBT and DBTBr, 330 nm.

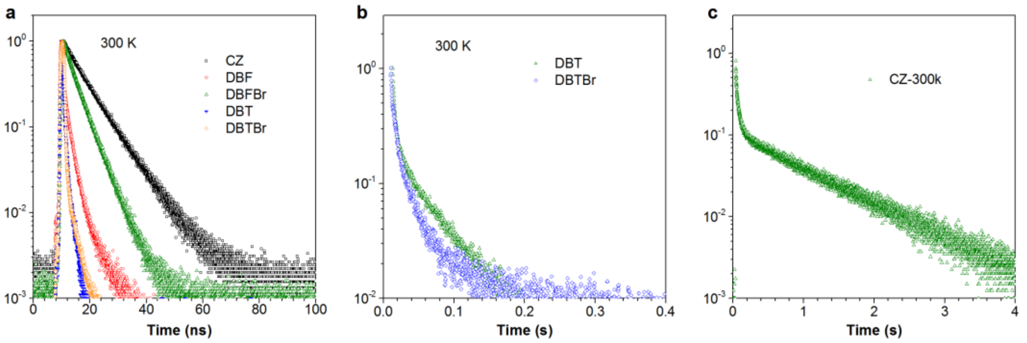

Supplementary Figure 28. Lifetime of RTP compounds in solid states at 4 K. (**a**) Nanosecond-scale PL decay curves of crystalline CZ, DBF, DBFBr DBT and DBTBr. (**b**, **c**) Second-scale PL decay curves of crystalline DBF, DBFBr, DBT, DBTBr (**b**) and CZ (**c**) at 4 K.

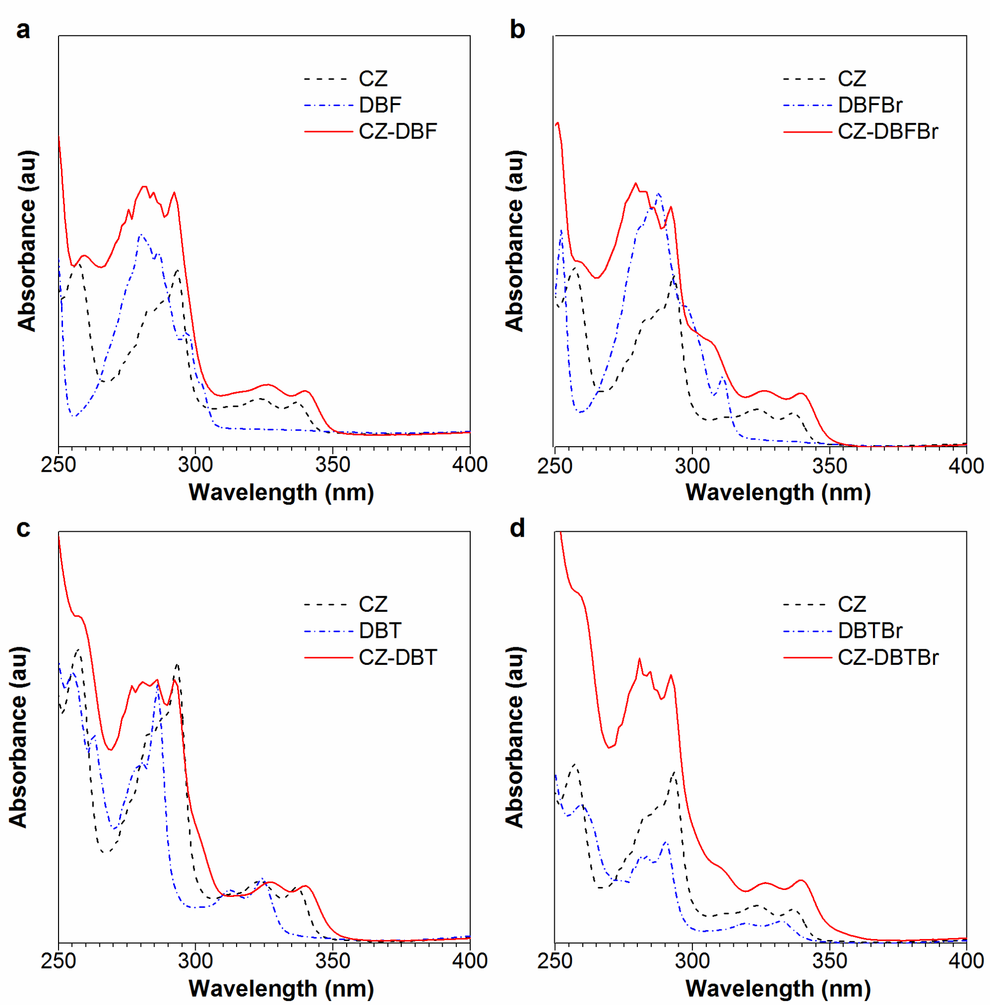

Supplementary Figure 29. UV-Vis spectra of RTP compounds in THF solutions. UV-Vis spectra of CZ, DBF and CZ-DBF (**a**), DBFBr and CZ-DBFBr (**b**), DBT and CZ-DBT (**c**), DBTBr and CZ-DBTBr (**d**). Concentration: 10^{-5} M.

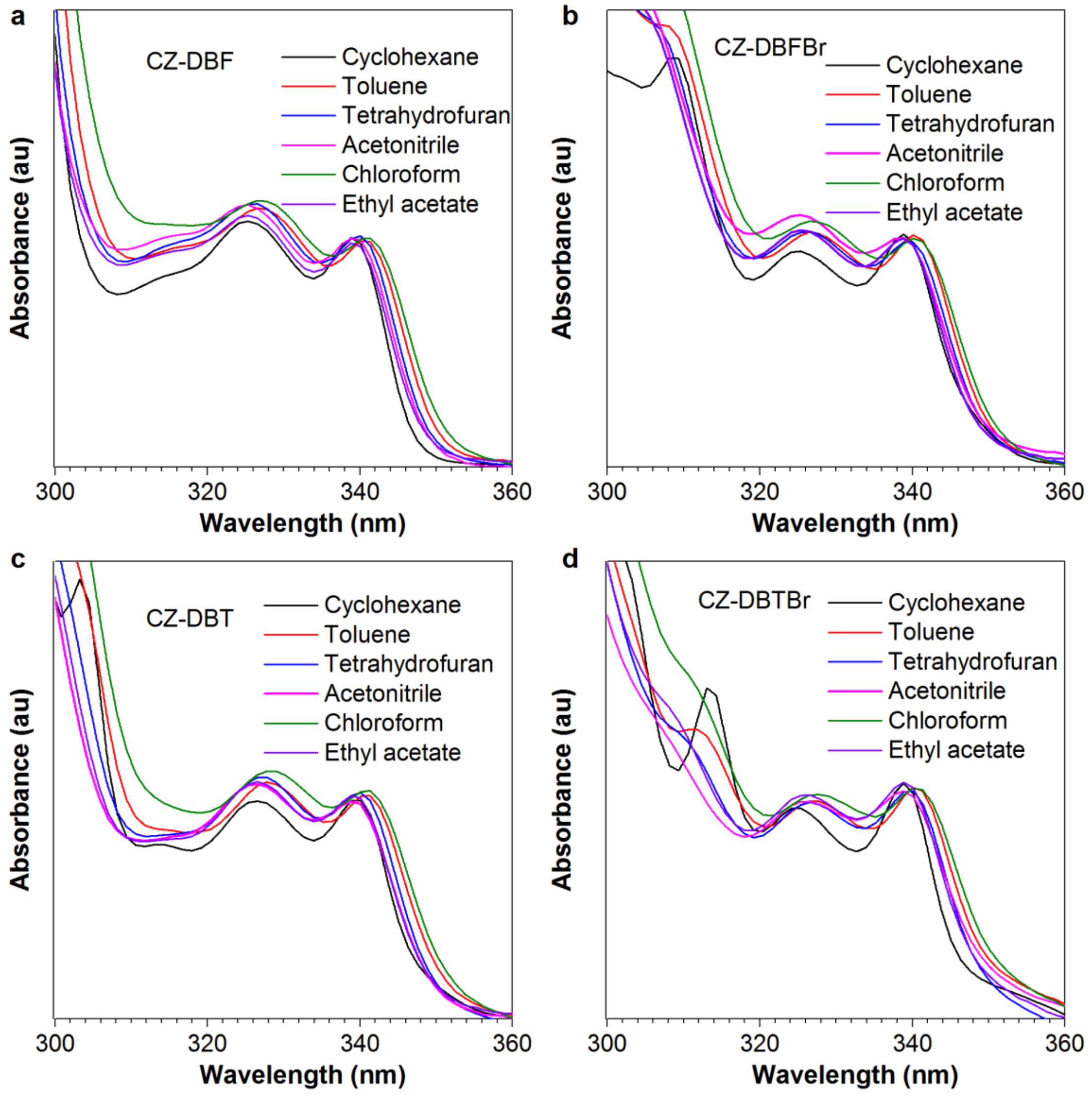

Supplementary Figure 30. UV-Vis spectra of OPRTP compounds in solutions. UV-Vis spectra of CZ-DBF (**a**), CZ-DBFBr (**b**), CZ-DBT (**c**) and CZ-DBTBr (**d**) in different solvents. Concentration: 10^{-5} M.

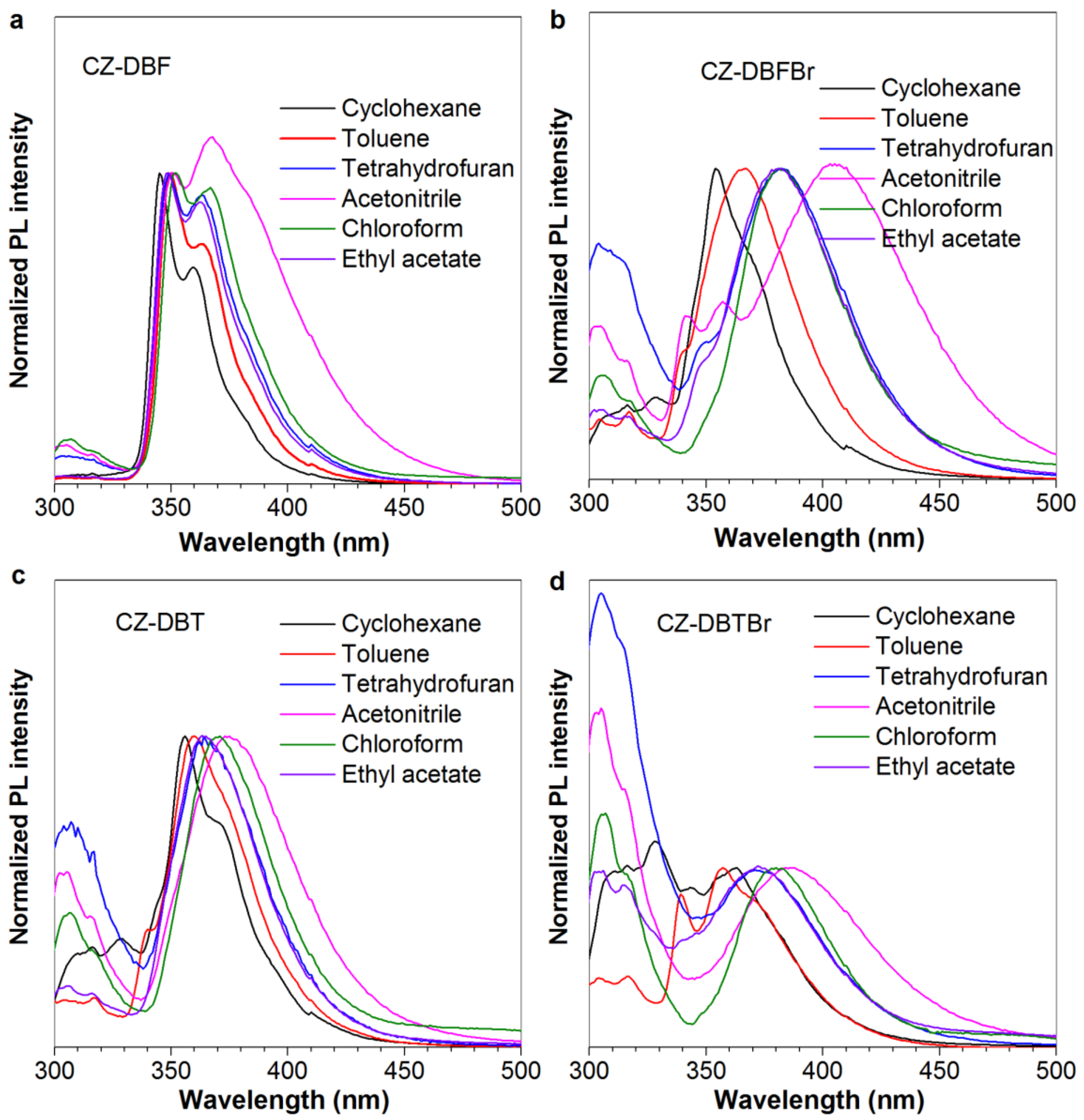

Supplementary Figure 31. PL spectra of OPRTP compounds in solutions. Normalized PL spectra of CZ-DBF (**a**), CZ-DBFBr (**b**), CZ-DBT (**c**) and CZ-DBTBr (**d**) in different solvents. Concentration: 10^{-5} M.

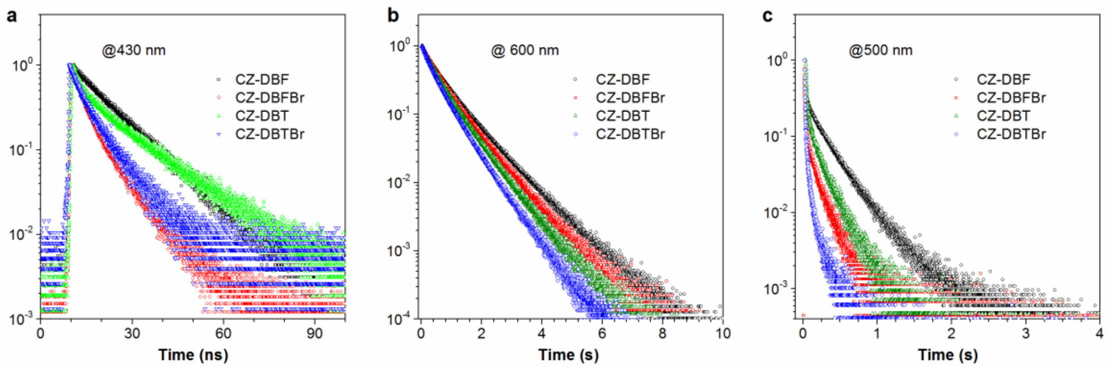

Supplementary Figure 32. The PL decay curves of OPRTP compounds at 4 K. (**a**) Nanosecond-scale PL decay curves measured at 430 nm. (**b**, **c**) Second-scale PL decay curves measured at 600 nm (**b**) and 500 nm (**c**) at 4 K.

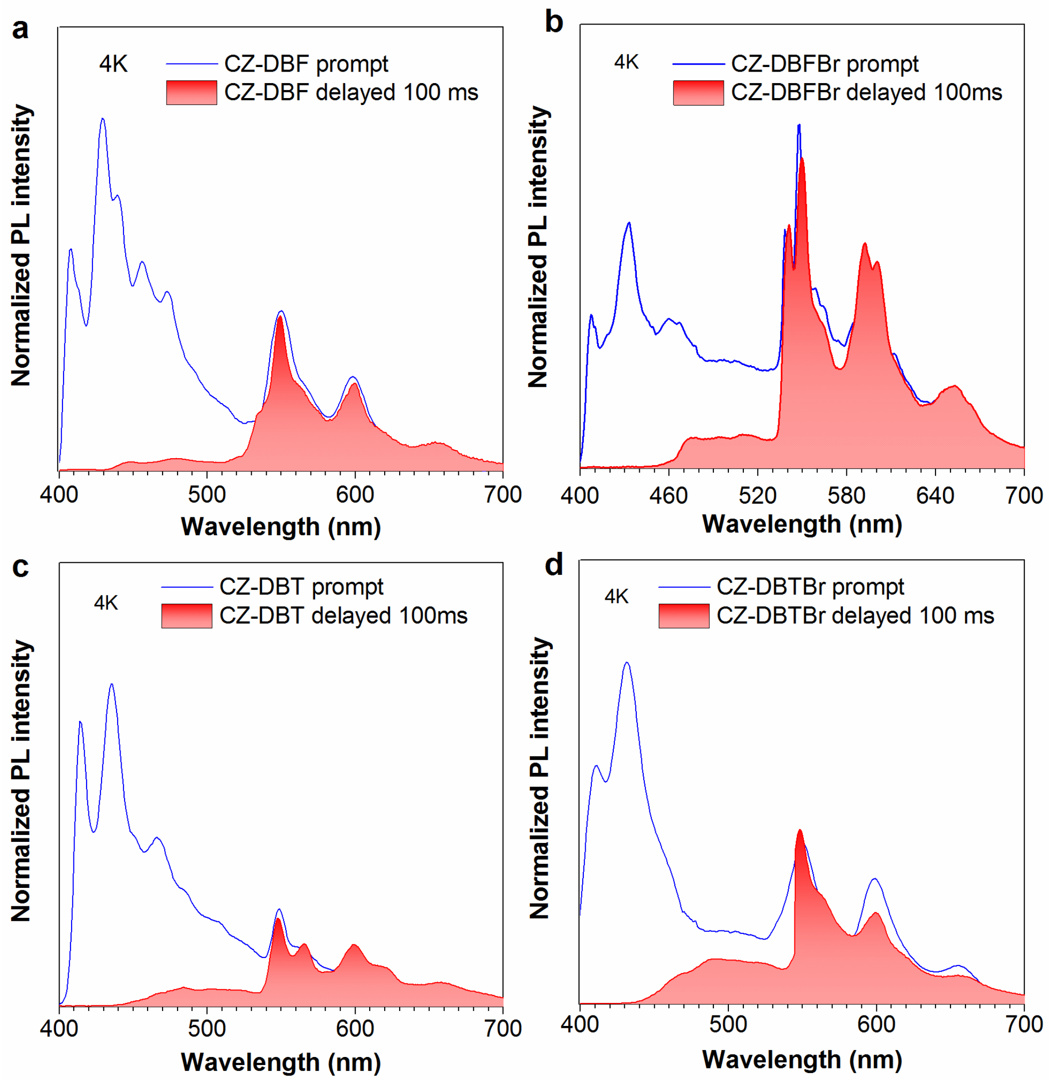

Supplementary Figure 33. PL spectra of crystalline OPRTP compounds at 4 K**.** The prompt (blue line) and delayed (red zone) PL spectra of the crystalline powders of CZ-DBF (**a**), CZ-DBFBr (**b**), CZ-DBT (**c**) and CZ-DBTBr (**d**) at 4 K. The excitation wavelength was 365 nm.

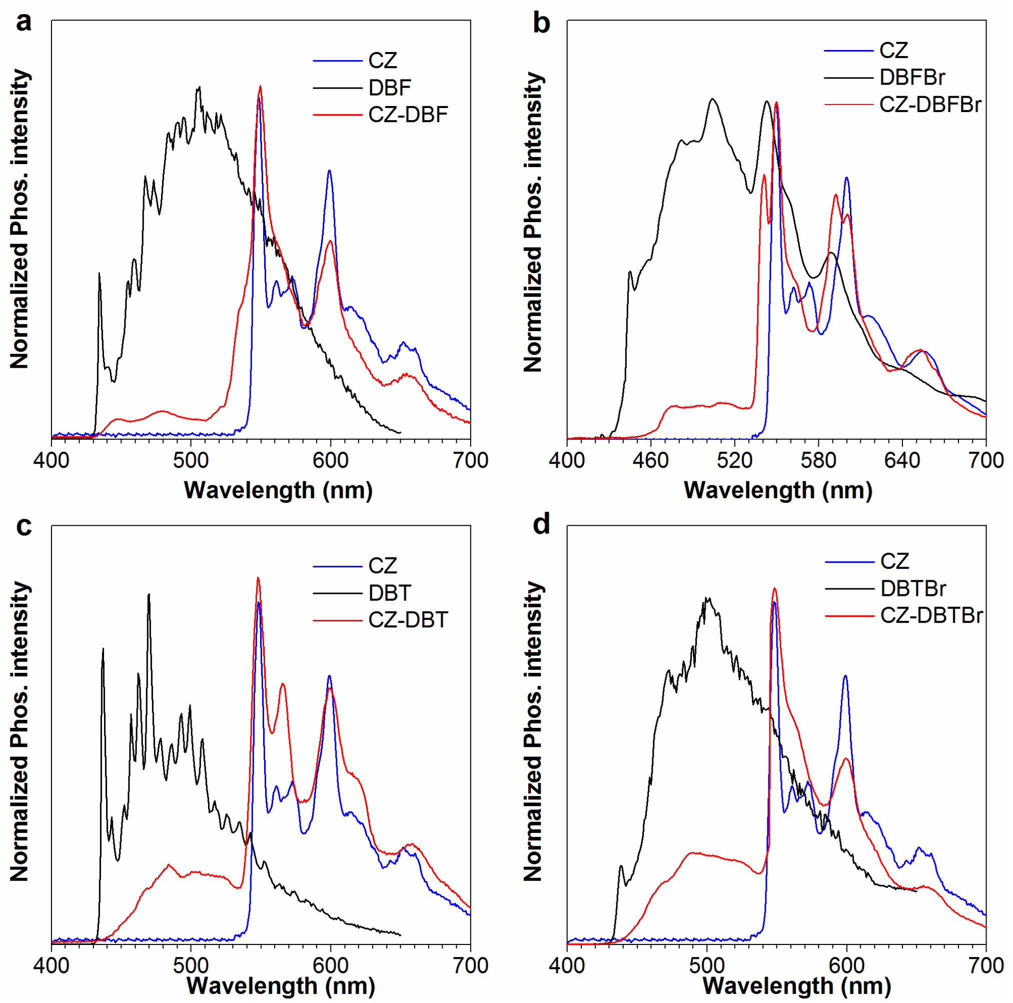

Supplementary Figure 34. PL spectra of crystalline all RTP compounds at 4 K**.** PL spectra of the crystalline powders of CZ and DBF (**a**), DBFBr (**b**), DBT (**c**) and DBTBr (**d**) at 4 K. Excitation wavelength: CZ, CZ-DBF, CZ-DBFBr, CZ-DBT and CZ-DBTBr, 365 nm; DBF, 295 nm; DBFBr, DBT and DBTBr, 330 nm.

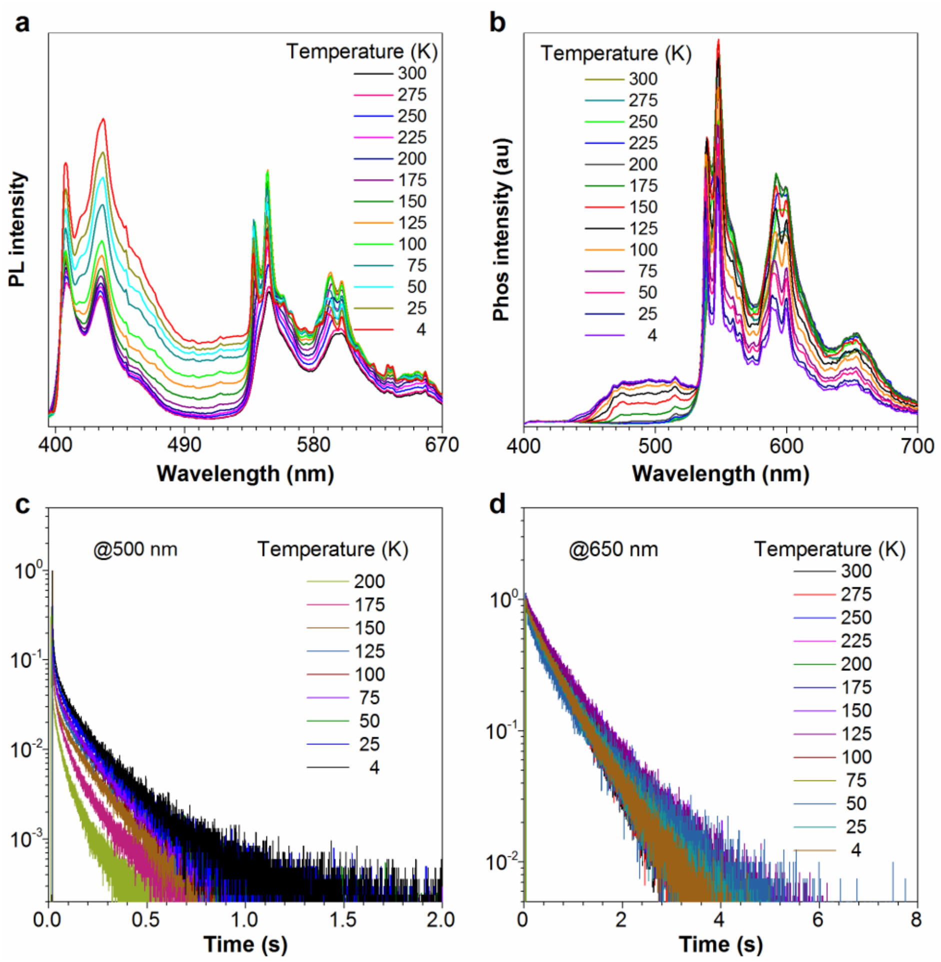

Supplementary Figure 35. Temperature dependent PL spectra and decay curves. The prompt (**a**), delayed PL (**b**) spectra and PL decay curves (**c**, **d**) of CZ-DBFBr measured at different temperatures from 4 to 300 K. a, Excited wavelength: 365 nm; b, c and d, Excited wavelength: 400 nm.

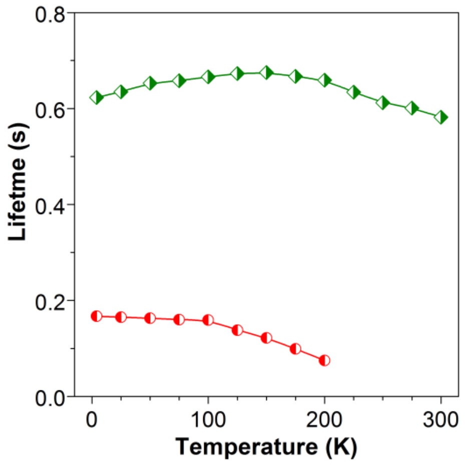

Supplementary Figure 36. Temperature-dependent lifetime of CZ-DBFBr. Temperature-dependent lifetime of emission bands at 500 (red line and symbol) and 655 nm (green line and symbol).

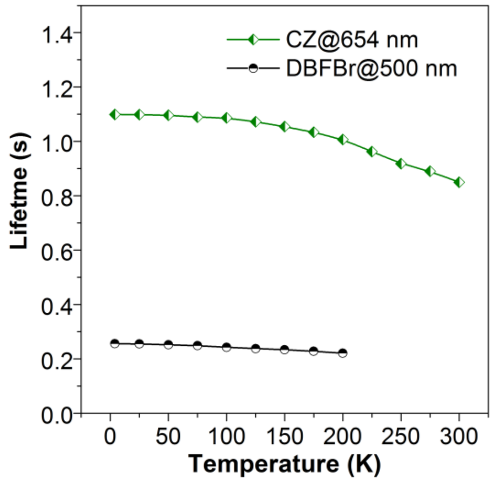

Supplementary Figure 37. Temperature-dependent lifetimes of CZ and BDFBr.

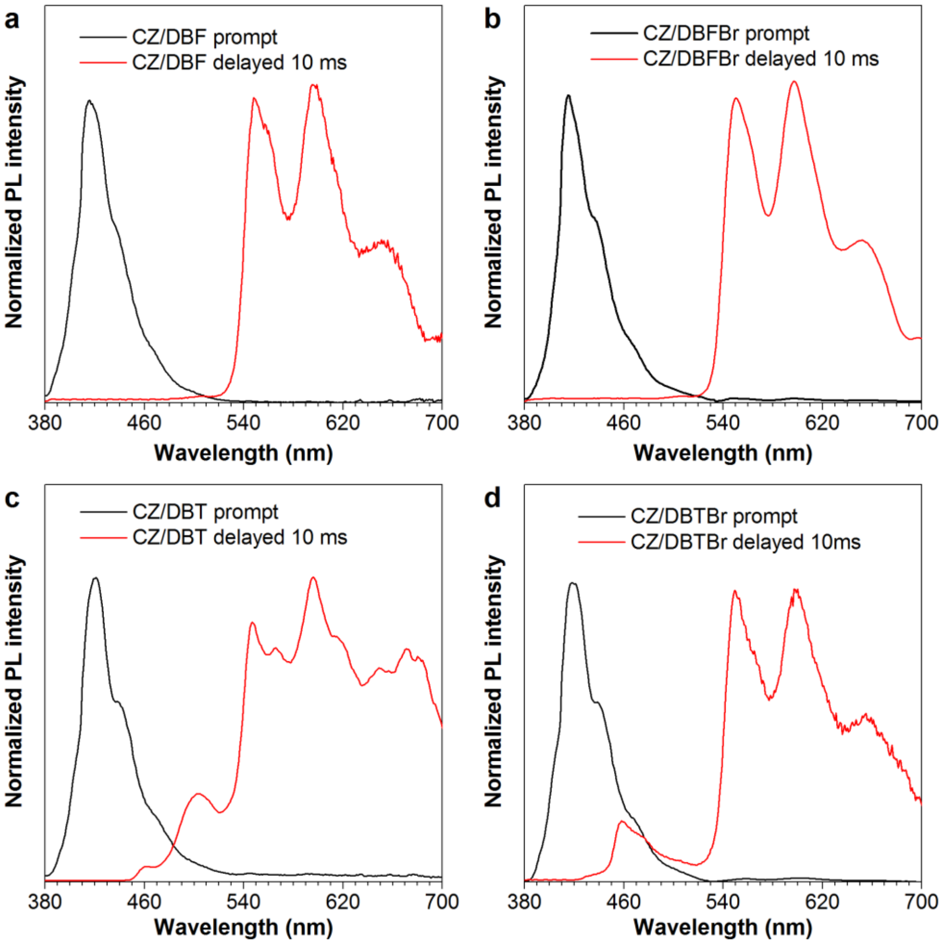

Supplementary Figure 38. PL spectra of crystalline blended model compounds**.** The prompt (black line) and delayed (red line) PL spectra of the blended powders of CZ and DBF (a), CZ and DBFBr (b), CZ and DBT (c), CZ and DBTBr (d) at 300 K. The excitation wavelength was 365 nm.

Supplementary Tables

Supplementary Table 1. Photophysical properties of crystalline model compounds. Photoluminescence wavelength (λ_F and λ_P), lifetime (τ_F and τ_P) and quantum efficiency (Φ_F and Φ_P) of crystalline DBF, DBFBr, DBT, DBTBr and CZ.

Compound	Temp.	λ_F (nm)	λ_P (nm)	τ_F (ns)	τ_P (ms)	Φ_F (%)	Φ_P (%)
DBF	4K	314[a]	435[a]	4.3	2344	4.1	-[d]
	300K	335[b]	-[c]	4.2	-[c]		
DBF-Br	4K	361[a]	440[a]	5.9	256	2.7	-[d]
	300K	380[b]	-[c]	4.6	-[c]		
DBT	4K	344[a]	437[a]	3.0	2870	1.6	0.32
	300K	362[b]	440[a]	2.3	55		
DBT-Br	4K	360[a]	439[a]	4.2	220	0.7	0.05
	300K	370[b]	441[a]	3.3	47		
CZ	4K	405[a]	549[a]	7.1	1110	78.2	-[d]
	300K	420[b]	550[b]	8.2	852		

[a] 0-0 peak of the photoluminescence wavelength.

[b] onset of the photoluminescence wavelength.

[c] the photoluminescence is too weak to be detected at room temperature.

[d] the photoluminescence is too weak to be detected at room temperature.

Supplementary Table 2. Photophysical properties of RTP compounds in solutions. Photoluminescence wavelength (λ_F and λ_P), phosphorescence lifetime (τ_P) of solution DBF, DBFBr, DBT,DBTBr, CZ-DBF, CZ-DBFBr, CZ-DBT and CZ-DBTBr in 2-methyl-tetrahydrofuran (10^{-5} M) measured at 77K.

Compound	λ_F (nm)	λ_P (nm)	τ_P (ms)
DBF	308[a]	402[a]	3807
DBF-Br	320[a]	410[a]	72
DBT	334[a]	411[a]	1122
DBT-Br	339[a]	414[a]	68
CZ	345[a]	406[a]	4512
CZ-DBF	344[b]	408[a]	4086
CZ-DBFBr	354[b]	409[a]	92
CZ-DBT	355[b]	411[a]	782
CZ-DBTBr	362[b]	415[a]	69

[a] 0-0 peak of the photoluminescence wavelength.
[b] onset of the photoluminescence wavelength.

Supplementary Table 3. Photophysical properties of crystalline OPRTP compounds Photoluminescence wavelength (λ_F and λ_P), lifetime (τ_F and τ_P) and quantum efficiency (Φ_F and Φ_P), rate constant of ISC (k_{isc}) of crystalline CZ-DBF, CZ-DBFBr, CZ-DBT and CZ-DBTBr.

Compound	Temp (K)	λ_F (nm)[a]	λ_P (nm)[a]	τ_F (ns)	τ_P (ms)	Φ_F (%)	Φ_P (%)	Φ_{isc} (%)	k_{isc} ($10^7 s^{-1}$)[b]
CZ-DBF	4	407	548	12.6	664	43.2	14.3	$56.8 > \Phi_{isc} > 14.3$	$4.51 > k_{isc} > 1.17$
	300	411	549	12.2	652				
CZ-DBFBr	4	407	548	8.1	598	31.8	41.2	$68.2 > \Phi_{isc} > 41.2$	$8.52 > k_{isc} > 5.15$
	300	411	550	8.0	540				
CZ-DBT	4	412	550	14.5	551	28.2	10.1	$71.8 > \Phi_{isc} > 10.1$	$5.84 > k_{isc} > 0.82$
	300	413	551	12.3	450				
CZ-DBTBr	4	412	550	8.2	490	22.3	12.1	$77.7 > \Phi_{isc} > 12.1$	$9.59 > k_{isc} > 1.49$
	300	413	551	8.1	420				

[a] 0-0 peak of the photoluminescence wavelength.

[b] see Supplementary Note 1.

Supplementary Table 4. Crystal Data and Structure Refinement for CZ-DBF.

Empirical formula	C24 H15 N O
Formula weight	333.37
Temperature	150(2) K
Wavelength	1.54184 Å
Crystal system	monoclinic
Space group	P1c1
Unit cell dimensions	a = 35.2958(11) Å alpha = 90 deg.
	b = 8.9222(3) Å beta = 95.2844(16) deg.
	c = 10.6378(3) Å gamma = 90 deg.
Volume	3335.78(18) $Å^3$
Z	8
Density (calculated)	1. 328 Mg/m^3
Absorption coefficient	0.634 mm^{-1}
F(000)	1392
Crystal size	0.20 x 0.12 x 0.08 mm^3
Theta range for data collection	3.773 to 74.173
Index ranges	-42<=h<=42, -10<=k<=10, -12<=l<=11
Reflections collected	11936
Independent reflections	9700[R(int) = 0.0548]
Absorption correction	Semi-empirical from equivalents
Max. and min. transmission	0.992 and 0.978
Refinement method	Full-matrix least-squares on F2
Data / restraints / parameters	9700 / 0 /937
Goodness-of-fit on F2	1.040
Final R indices [I>2sigma(I)]	R1 = 0.0661, wR2 = 0.1610
R indices (all data)	R1 = 0.0629, wR2 = 0.1634
Largest diff. peak and hole	0.526 and -0.329 $e.Å^{-3}$

Supplementary Table 5. Crystal Data and Structure Refinement for CZ-DBFBr.

Empirical formula	C24 H14 Br N O
Formula weight	412.27
Temperature	150 (2) K
Wavelength	1.54184 Å
Crystal system	monoclinic
Space group	P121/c1
Unit cell dimensions	a = 16.6644(8) Å alpha = 90 deg.
	b = 5.4635(3) Å beta = 92.8114(19) deg.
	c = 19.3068(9) Å gamma = 90 deg.
Volume	1755.69(15) $Å^3$
Z	4
Density (calculated)	1.560 Mg/m^3
Absorption coefficient	3.291 mm^{-1}
F(000)	832
Crystal size	0.10 x 0.10 x 0.05 mm^3
Theta range for data collection	4.586 to 74.856
Index ranges	-18<=h<=19, -6<=k<=6, -22<=l<=23
Reflections collected	25386
Independent reflections	3119 [R(int) = 0.0768]
Absorption correction	Semi-empirical from equivalents
Max. and min. transmission	0.982 and 0.9954
Refinement method	Full-matrix least-squares on F2
Data / restraints / parameters	3119 / 0 /244
Goodness-of-fit on F2	1.208
Final R indices [I>2sigma(I)]	R1 = 0.0742, wR2 = 0.1835
R indices (all data)	R1 = 0.0718, wR2 = 0.1848
Largest diff. peak and hole	1.777 and -0.999 e.$Å^{-3}$

Supplementary Table 6. Crystal Data and Structure Refinement for CZ-DBT.

Empirical formula	C24 H15 N O
Formula weight	349.09
Temperature	100(10) K
Wavelength	1.54184 Å
Crystal system	orthorhombic
Space group	P212121
Unit cell dimensions	a = 5.9335(2) Å alpha = 90 deg.
	b = 16.5651(6) Å beta = 90 deg.
	c = 35.3274(10) Å gamma = 90 deg.
Volume	3472.3(2) $Å^3$
Z	4
Density (calculated)	1. 279 Mg/m^3
Absorption coefficient	1.668 mm^{-1}
F(000)	1336
Crystal size	0.40 x 0.30 x 0.20 mm^3
Theta range for data collection	1.95 to 27.58
Index ranges	-6<=h<=7, -16<=k<=19, -42<=l<=42
Reflections collected	19992
Independent reflections	6171 [R(int) = 0.0704]
Absorption correction	Semi-empirical from equivalents
Max. and min. transmission	0.995 and 0.995
Refinement method	Full-matrix least-squares on F2
Data / restraints / parameters	6171/ 0 /469
Goodness-of-fit on F2	1.001
Final R indices [I>2sigma(I)]	R1 = 0.0598, wR2 = 0.1486
R indices (all data)	R1 = 0.0828, wR2 = 0.1657
Largest diff. peak and hole	1.011 and -0.219 $e.Å^{-3}$

Supplementary Notes

Supplementary Note 1. Calculation of photophysical parameters of OPRTP .

Theoretically, the lifetime (τ_F and τ_P) and photoluminescence quantum yield (Φ_F and Φ_P) of fluorescence and RTP as well as quantum yield of ISC (Φ_{ISC}) can be expressed as Supplementary Equation 1-5:

$$\tau_F = 1/\left(k_F + k_{nr}^F + k_{ISC}\right) \quad (1)$$

$$\tau_P = 1/\left(k_P + k_{nr}^P\right) \quad (2)$$

$$\Phi_F = k_F/\left(k_F + k_{nr}^F + k_{ISC}\right) = k_F \times \tau_F \quad (3)$$

$$\Phi_P = \Phi_{ISC} \times k_P/\left(k_P + k_{nr}^P\right) = \Phi_{ISC} \times k_P \times \tau_P \quad (4)$$

$$\Phi_{ISC} = k_{ISC}/\left(k_{ISC} + k_F + k_{nr}^F\right) = k_{ISC} \times \tau_F \quad (5)$$

We can easily obtain Supplementary Equation 6 from Supplementary Equation 2:

$$\Phi_{ISC} = \Phi_P(1 + k_{nr}^P/k_P) \quad (6)$$

From Supplementary Equation 6, we know that the value of Φ_{ISC} should be higher than Φ_P but lower than $1-\Phi_F$. Therefore, k_{ISC} can be estimated by Φ_{ISC}/τ_F accordingly.

Supplementary References

1 Sun, X. *et al*. External Heavy-Atom Effect via Orbital Interactions Revealed by Single-Crystal X-ray Diffraction. *J. Phys. Chem. A* **120**, 5791-5797 (2016).

2 Zhang, T. *et al*. Pure Organic Persistent Room-Temperature Phosphorescence at both Crystalline and Amorphous States. *ChemPhysChem.* **19**, 2389-2396 (2018).

3 An, Z. *et al*. Stabilizing triplet excited states for ultralong organic phosphorescence. *Nat. Mater.* **14**, 685-690 (2015).

4 Xie, Y. *et al*. How the Molecular Packing Affects the Room Temperature Phosphorescence in Pure Organic Compounds: Ingenious Molecular Design, Detailed Crystal Analysis, and Rational Theoretical Calculations. *Adv. Mater.* **29**, 1606829 (2017).

5 Xue, P. *et al.* Luminescence switching of a persistent room-temperature phosphorescent pure organic molecule in response to external stimuli. *Chem. Commun.* **51**,10381-10384 (2015).

6 Li, C. *et al.* Reversible Luminescence Switching of an Organic Solid: Controllable On-Off Persistent Room Temperature Phosphorescence and Stimulated Multiple Fluorescence Conversion. *Adv. Opt. Mater.* **3**,

1184-1190 (2015).
7 Fateminia, SMA. *et al.* Organic Nanocrystals with Bright Red Persistent Room-Temperature Phosphorescence for Biological Applications. *Angew. Chem. Int. Ed.* **56**, 12160-12164 (2017).
8 Yang, Z. *et al.* Intermolecular Electronic Coupling of Organic Units for Efficient Persistent Room-Temperature Phosphorescence. *Angew. Chem. Int. Ed.* **55**, 2181-2185 (2016).
9 Cai, S. *et al.* Enhancing Ultralong Organic Phosphorescence by Effective π-Type Halogen Bonding. *Adv. Funct. Mater.* **28**, 1705045 (2018).
10 Gong, Y. *et al.* Achieving Persistent Room Temperature Phosphorescence and Remarkable Mechanochromism from Pure Organic Luminogens. *Adv. Mater.* **27**, 6195-6201 (2015).
11 Xiong, Y. *et al.* Designing Efficient and Ultralong Pure Organic Room-Temperature Phosphorescent Materials by Structural Isomerism. *Angew. Chem. Int. Ed.* **57**, 7997-8001 (2018).
12 Gu, L. *et al.* Prolonging the lifetime of ultralong organic phosphorescence through dihydrogen bonding. *J. Mater. Chem. C* **6**, 226-233 (2018).
13 Mu, Y. *et al.* Mechano-induced persistent room-temperature phosphorescence from purely organic molecules. *Chem. Sci.* **9**, 3782-3787 (2018).
14 Zhang, K. *et al.* Cyclic boron esterification: screening organic room temperature phosphorescent and mechanoluminescent materials. *J. Mater. Chem. C* **6**, 8733-8737 (2018).
15 Sun, C. *et al.* Twisted Molecular Structure on Tuning Ultralong Organic Phosphorescence. *J. Phys. Chem. Lett.* **9**, 335-339 (2018).
16 Xu, L. *et al.* Chalcogen atom modulated persistent room-temperature phosphorescence through intramolecular electronic coupling. *Chem. Commun.* **54**, 9226-9229 (2018).
17 Cai, S. *et al.* Visible-Light-Excited Ultralong Organic Phosphorescence by Manipulating Intermolecular Interactions. *Adv. Mater.* **29**, 1701244 (2017).
18 Mao, Z. *et al.* The methylation effect in prolonging the pure organic room temperature phosphorescence lifetime. *Chem. Sci.* doi:10.1039/C8SC03019G (2018).
19 Gu, L. *et al.* Dynamic Ultralong Organic Phosphorescence by Photoactivation. *Angew. Chem. Int. Ed.* **57**, 8425-8431 (2018).
20 Zhang, Z. *et al.* N-Alkylcarbazoles: homolog manipulating long-lived room-temperature phosphorescence. *J. Mater. Chem. C* **6**, 8984-8989 (2018).
21 He, Z. *et al.* White light emission from a single organic molecule with dual

phosphorescence at room temperature. *Nat. Commun.* **8**, 416 (2017).
22 Lucenti, E. *et al.* H-Aggregates Granting Crystallization-Induced Emissive Behavior and Ultralong Phosphorescence from a Pure Organic Molecule. *J. Phys. Chem. Lett.* **8**, 1894-1898 (2017).
23 Lucenti, E. *et al.* Cyclic Triimidazole Derivatives: Intriguing Examples of Multiple Emissions and Ultralong Phosphorescence at Room Temperature. *Angew. Chem. Int. Ed.* **56**, 16302-16307 (2017).
24 Kuno, S. *et al.* Visible room-temperature phosphorescence of pure organic crystals via a radical-ion-pair mechanism. *Phys. Chem. Chem. Phys.* **17**, 15989-15995 (2015).
25 Cheng, Z. *et al.* Ultralong Phosphorescence from Organic Ionic Crystals under Ambient Conditions. *Angew. Chem. Int. Ed.* **57**, 678-682 (2018).
26 Kuno, S. *et al.* Long Persistent Phosphorescence of Crystalline Phenylboronic Acid Derivatives: Photophysics and a Mechanistic Study. *ChemPhotoChem* **1**, 102-106 (2017).
27 Chai, Z. *et al.* Abnormal room temperature phosphorescence of purely organic boron-containing compounds: the relationship between the emissive behavior and the molecular packing, and the potential related applications. *Chem. Sci.* **8**, 8336-8344 (2017).
28 Zhao, W. *et al.* Rational Molecular Design for Achieving Persistent and Efficient Pure Organic Room-Temperature Phosphorescence. *Chem* **1**, 592-602 (2016).
29 Shoji, Y. *et al.* Unveiling a New Aspect of Simple Arylboronic Esters: Long-Lived Room-Temperature Phosphorescence from Heavy-Atom-Free Molecules. *J. Am. Chem. Soc.* **139**, 2728-2733 (2017).
30 Tao, Y. *et al.* Resonance-Activated Spin-Flipping for Efficient Organic Ultralong Room-Temperature Phosphorescence. *Adv. Mater.* **30**, 1803856 (2018).
31 Ma, C. *et al.* Insight into chirality on molecular stacking for tunable ultralong organic phosphorescence. *J. Mater. Chem. C* **6**, 10179-10183 (2018).
